# Surface engineering on a microporous metal–organic framework to boost ethane/ethylene separation under humid conditions†

**DOI:** 10.1039/d3sc04119k

**Published:** 2023-10-09

**Authors:** Xiao-Jing Xie, Ying Wang, Qi-Yun Cao, Rajamani Krishna, Heng Zeng, Weigang Lu, Dan Li

**Affiliations:** a College of Chemistry and Materials Science, Guangdong Provincial Key Laboratory of Functional Supramolecular Coordination Materials and Applications, Jinan University Guangzhou 510632 P. R. China zengheng90@163.com weiganglu@jnu.edu.cn; b Van't Hoff Institute for Molecular Sciences, University of Amsterdam Science Park 904 Amsterdam 1098 XH Netherlands

## Abstract

Recently, examples of metal–organic frameworks (MOFs) have been identified displaying ethane (C_2_H_6_) over ethylene (C_2_H_4_) adsorption selectivity. However, it remains a challenge to construct MOFs with both large C_2_H_6_ adsorption capacity and high C_2_H_6_/C_2_H_4_ adsorption selectivity, especially under humid conditions. Herein, we reported two isoreticular MOF-5 analogues (JNU-6 and JNU-6-CH_3_) and their potential applications in one-step separation of C_2_H_4_ from C_2_H_6_/C_2_H_4_ mixtures. The introduction of CH_3_ groups not only reduces the pore size from 5.4 Å in JNU-6 to 4.1 Å in JNU-6-CH_3_ but also renders an increased electron density on the pyrazolate N atoms of the organic linker. JNU-6-CH_3_ retains its framework integrity even after being immersed in water for six months. More importantly, it exhibits large C_2_H_6_ adsorption capacity (4.63 mmol g^−1^) and high C_2_H_6_/C_2_H_4_ adsorption selectivity (1.67) due to the optimized pore size and surface function. Breakthrough experiments on JNU-6-CH_3_ demonstrate that C_2_H_4_ can be directly separated from C_2_H_6_/C_2_H_4_ (50/50, v/v) mixtures, affording benchmark productivity of 22.06 and 18.71 L kg^−1^ of high-purity C_2_H_4_ (≥99.95%) under dry and humid conditions, respectively.

## Abstract 2

A linker methylation strategy to tune the pore size and surface electrostatic potential, rendering JNU-6-CH_3_ capable of directly separating high-purity C_2_H_4_ from C_2_H_6_/C_2_H_4_ mixtures with benchmark separation capacity under dry or humid conditions.

## Graphical abstract

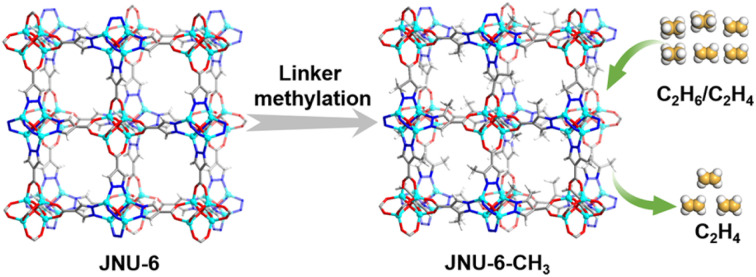

As one of the seven world-changing chemical separations, olefin/paraffin separation accounts for more than 0.3% of global energy consumption.^1^ Ethylene (C_2_H_4_) is an important chemical feedstock in petrochemical industries, with a global production capacity of over 200 million tons in 2023.^2^ At present, C_2_H_4_ is mainly produced *via* steam cracking of ethane (C_2_H_6_) in industry, which would inevitably leave a certain amount of C_2_H_6_ in the obtained C_2_H_4_. Given that the C_2_H_6_ impurity may interfere with the polymerization process, further purification is required and the polymer-grade (≥99.95%) C_2_H_4_ is highly desired in the manufacture of value-added chemicals. Owing to their very similar physicochemical properties and molecular sizes (3.81 × 4.08 × 4.82 Å^3^ and 3.28 × 4.18 × 4.84 Å^3^ for C_2_H_6_ and C_2_H_4_, respectively), the industrial C_2_H_4_/C_2_H_6_ separation relies on cryogenic distillation, which is energy intensive and requires high distillation towers with many trays in order to achieve high reflux ratios.^3^

Compared to traditional distillation, non-thermal separation technologies using porous materials are of great significance to energy-efficient separation economy.^4,5^ Metal–organic frameworks (MOFs), also known as porous coordination polymers (PCPs),^6–8^ have been extensively investigated in hydrocarbon separation due to their highly tunable pore geometry and surface chemistry. With regard to C_2_H_4_/C_2_H_6_ separation, MOFs can be categorized into two types: C_2_H_6_-selective and C_2_H_4_-selective. For C_2_H_4_-selective MOFs, desorption by heat or purge is necessary in order to obtain C_2_H_4_, which likely would result in C_2_H_6_ contamination. For example, the benchmark C_2_H_4_/C_2_H_6_ sieving MOF, UTSA-280,^9^ can realize complete exclusion of large-sized C_2_H_6_ molecules and an infinite C_2_H_4_ over C_2_H_6_ selectivity, yet C_2_H_4_ with only 99.1% purity was reported upon desorption.

By contrast, C_2_H_6_-selective MOFs allow for direct production of C_2_H_4_ in a single adsorption step, which could potentially save *ca.* 40% of energy consumption (0.4 to 0.6 GJ ton^−1^ of C_2_H_4_) for C_2_H_4_/C_2_H_6_ separation.^10^ Considering the numbers of hydrogen atoms on the surface of C_2_H_6_ and C_2_H_4_ molecules (6 *vs.* 4), controlled surface engineering with polar functions (*e.g.*, N- and O-containing groups) on the pore walls may facilitate non-classic hydrogen bonding and stronger affinity toward C_2_H_6_ than C_2_H_4_.^5,11–16^ Nevertheless, water vapor may compete for the interactions with those polar functional groups, leading to substantially reduced separation potential under humid conditions. For example, the benchmark C_2_H_6_-selective MOF, Fe_2_(O_2_)(dobdc),^17^ demonstrated an excellent C_2_H_6_ over C_2_H_4_ selectivity with a record separation factor of *ca.* 4.4. The material itself, however, is extremely sensitive to moisture and has to be handled in a glove box. Recent studies show that nonpolar pore environments can prevent moisture from entering inside the frameworks and therefore retain the C_2_H_4_/C_2_H_6_ separation potential even under humid conditions. More importantly, nonpolar pore surfaces may still facilitate C_2_H_6_ over C_2_H_4_ selectivity due to their slightly different polarizability (C_2_H_6_: 44.7 × 10^25^ cm^3^, C_2_H_4_: 42.5 × 10^25^ cm^3^). For instance, the MOF FJI-H11-Me-(des),^18^ featuring nonpolar pore surfaces comprised of aromatic rings and alkyl groups, exhibits a stable C_2_H_6_ over C_2_H_4_ separation capacity in a wide range of relative humidities (RHs). However, the overall separation potential was limited due to its moderate adsorption capacity for C_2_H_6_ (2.58 mmol g^−1^). Until now, it remains a challenge to construct MOFs with both large C_2_H_6_ adsorption capacity and high C_2_H_6_ over C_2_H_4_ adsorption selectivity to break the adsorption/selectivity trade-off limitation, especially under humid conditions.

Isoreticular chemistry allows for the design and synthesis of MOFs with tailor-made pore dimensions and functions for selective binding of one over the other in C_2_H_4_/C_2_H_6_ separation. The methyl (CH_3_) group is electron-donating, and its effect on gas adsorption and separation has been well documented.^19–21^ In addition, the CH_3_ group is considered strongly hydrophobic, and the MOF decorated with CH_3_ groups usually exhibits low water adsorption capacity even at high RH, which could effectively suppress the competition of water vapor for adsorption sites. Herein, we selected Zn_4_O(PyC)_3_ (termed here as JNU-6, H_2_PyC = pyrazole-4-carboxylic acid), an isoreticular MOF-5 analogue,^22–24^ as the platform for surface functionalization *via* linker methylation. We found that the introduction of CH_3_ groups not only reduces the pore size from 5.4 Å in JNU-6 to 4.1 Å in JNU-6-CH_3_ but also renders an increased electron density on the pyrazolate N atoms of the organic linker. As a result, JNU-6-CH_3_ retains its framework integrity even after being immersed in water for six months. More importantly, it exhibits large C_2_H_6_ adsorption capacity (4.63 mmol g^−1^) and high C_2_H_6_/C_2_H_4_ adsorption selectivity (1.67) due to the optimized pore size and surface function. Breakthrough experiments on JNU-6-CH_3_ demonstrate benchmark productivity of 22.06 and 18.71 L kg^−1^ of high-purity C_2_H_4_ (≥99.95%) from a C_2_H_6_/C_2_H_4_ (50 : 50) mixture under dry and humid conditions, respectively.

To apply reticular chemistry and address the separation challenge of C_2_H_6_/C_2_H_4_ under humid conditions, it is crucial to find a C_2_H_6_-selective MOF that can be easily functionalized. In this work, we selected an isoreticular analogue of MOF-5 as the platform for the introduction of CH_3_ groups. A solvothermal reaction of Zn(NO_3_)_2_ with pyrazole-4-carboxylic acid or 3-methylpyrazole-4-carboxylic acid in a mixed solution of DEF/H_2_O afforded high-quality block crystals of JNU-6 and JNU-6-CH_3_, respectively. Single crystal X-ray diffraction (SCXRD) analyses reveal that JNU-6 and JNU-6-CH_3_ are of cubic crystal structure isoreticular to MOF-5. It should be pointed out that both JNU-6 and JNU-6-CH_3_ were reported by Zhong and co-workers recently for *n*-C_4_H_10_/iso-C_4_H_10_ separation during our preparation of this paper.^25^ In the crystal structures, two types of octahedral Zn_4_O SBUs (secondary building units, Zn_4_ON_12_ and Zn_4_O(COO)_6_) are connected by ditopic organic linkers to form a 3-dimensional (3D) network with interconnected cubic-shaped cages (Fig. 1a). The introduction of CH_3_ groups on the pore surface decreases the aperture size from 5.4 Å to 4.1 Å (Fig. 1b), making it more comparable to the kinetic diameters of C_2_H_6_ and C_2_H_4_ (C_2_H_6_ = 4.44 Å, C_2_H_4_ = 4.16 Å).^26^ Density functional theory (DFT) calculations were carried out to generate the mapping of electrostatic potential (ESP) on JNU-6 and JNU-6-CH_3_. As shown in Fig. 1c, an increased electron density was observed on the pyrazole rings of JNU-6-CH_3_, particularly around the N atoms, owing to the electron-donating effect of the CH_3_ groups. Such an electrostatic potential in JNU-6-CH_3_ indicates an increased surface dipole, which may potentially facilitate the discrimination of C_2_H_6_ from C_2_H_4_ due to their slightly different polarizability.

**Fig. 1:**
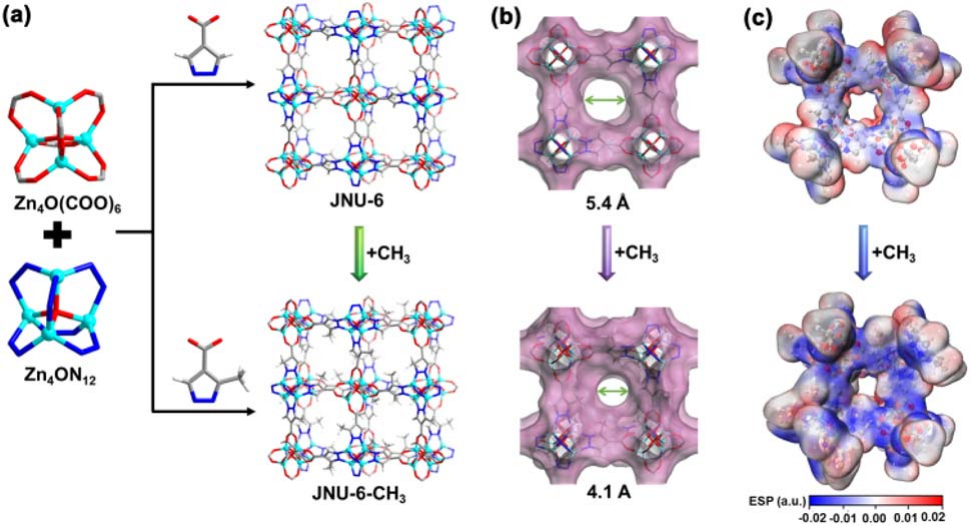
(a) Isostructural frameworks of JNU-6 and JNU-6-CH_3_ assembled with two six-connected Zn_4_O SBUs and their respective organic linkers. (Color code: Zn, cyan; C, dark gray; N, blue; O, red; H, white). (b) Connolly surface analysis of JNU-6 and JNU-6-CH_3_, depicting the reduced pore size upon the introduction of CH_3_ groups. (c) Electrostatic potential mapping of JNU-6 and JNU-6-CH_3_, depicting the increased electron density on pyrazolate N atoms upon the introduction of CH_3_ groups.

The phase purity and crystallinity of the bulk JNU-6 and JNU-6-CH_3_ samples were checked by powder X-ray diffraction (PXRD) analyses, showing good agreement with the ones simulated from their respective crystal structures. N_2_ adsorption/desorption isotherms at 77 K were measured to investigate the porosity of JNU-6 and JNU-6-CH_3_. As shown in Fig. 2a, both of them exhibit type-I adsorption/desorption isotherms characteristic of microporous materials. Due to the introduction of CH_3_ groups, the calculated Brunauer–Emmett–Teller (BET) surface area of JNU-6-CH_3_ is slightly decreased from 1411 m^2^ g^−1^ in JNU-6 to 1270 m^2^ g^−1^, and the calculated pore volume is also decreased from 0.59 cm^3^ g^−1^ in JNU-6 to 0.51 cm^3^ g^−1^. Further, the pore size distribution was determined by the Horvath–Kawazoe model and the dominant pore diameters exhibit the same trend, with values decreasing from 5.4 Å in JNU-6 to 4.1 Å in JNU-6-CH_3_ (Fig. 2a, inset).

**Fig. 2:**
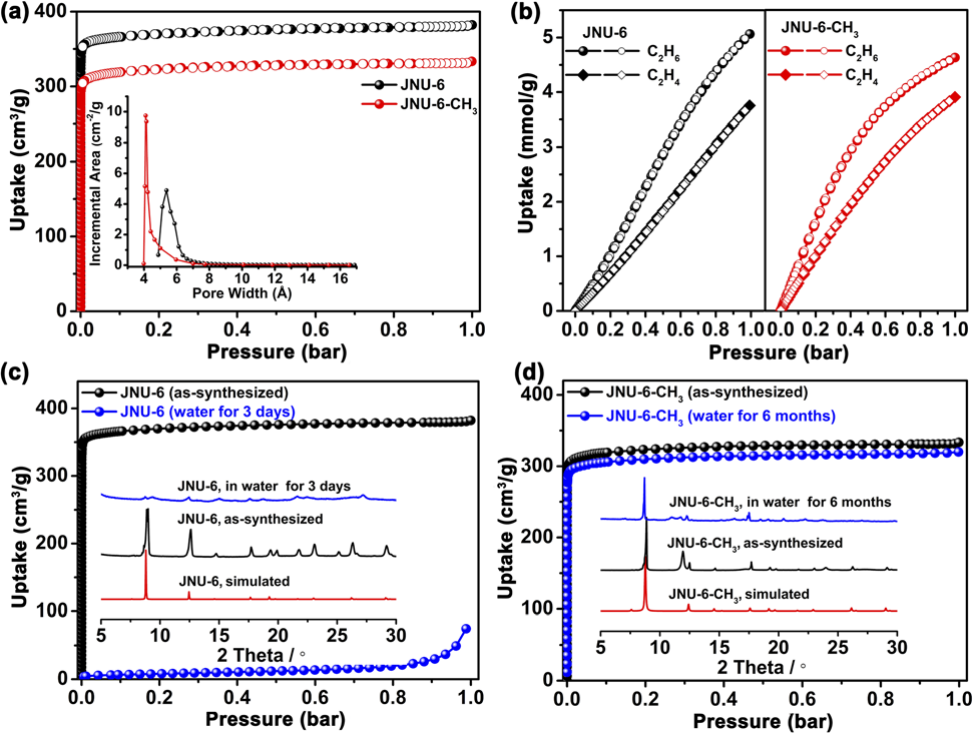
(a) N_2_ adsorption/desorption isotherms of JNU-6 and JNU-6-CH_3_ at 77 K. Inset shows the difference in their pore size distribution. (b) C_2_H_6_ and C_2_H_4_ adsorption/desorption isotherms of JNU-6 and JNU-6-CH_3_ at 298 K. (c) Comparison of N_2_ adsorption isotherms at 77 K and PXRD patterns of the as-synthesized JNU-6 and water-treated JNU-6 (being soaked in water for 3 days). (d) Comparison of N_2_ adsorption isotherms at 77 K and PXRD patterns of the as-synthesized JNU-6-CH_3_ and water-treated JNU-6-CH_3_ (being soaked in water for 6 months).

Single-component adsorption isotherms of JNU-6 and JNU-6-CH_3_ for C_2_H_6_ and C_2_H_4_ were measured at 298 K. As exhibited in Fig. 2b, the C_2_H_6_ adsorption capacity is substantially larger than C_2_H_4_ in the entire pressure range (0–1 bar) for both JNU-6 and JNU-6-CH_3_. The maximum uptakes for C_2_H_4_ are 84.4 cm^3^ g^−1^ (3.77 mmol g^−1^) and 88.1 cm^3^ g^−1^ (3.93 mmol g^−1^) on JNU-6 and JNU-6-CH_3_, respectively, while the uptakes for C_2_H_6_ can reach up to 113.6 cm^3^ g^−1^ (5.07 mmol g^−1^) and 103.7 cm^3^ g^−1^ (4.63 mmol g^−1^) on JNU-6 and JNU-6-CH_3_, respectively. The C_2_H_6_ uptakes on JNU-6 and JNU-6-CH_3_ are comparable to or larger than those of most of the C_2_H_6_-selective MOFs, such as Cu(Qc)_2_ (1.84 mmol g^−1^),^27^ MUF-15 (4.69 mmol g^−1^),^28^ NKMOF-8-Br (4.22 mmol g^−1^),^29^ FJI-H11-Me-(des) (2.58 mmol g^−1^),^18^ Ni(IN)_2_ (3.05 mmol g^−1^),^30^ AzoleTh-1 (4.47 mmol g^−1^),^31^ and NPU-1 (4.5 mmol g^−1^).^32^ We applied the ideal adsorbed solution theory (IAST) to calculate the adsorption selectivity, and the IAST selectivity of JNU-6-CH_3_ for a C_2_H_6_/C_2_H_4_ (50 : 50) mixture at 298 K can reach up to 1.67 (Fig. S4–S9†), which is comparable to those of the reported benchmark MOF adsorbents, such as MUF-15 (1.96),^28^ NKMOF-8-Br (2.65),^29^ NKMOF-8-Me (1.88),^29^ Ni(IN)_2_ (2.44),^30^ AzoleTh-1 (1.46),^31^ and NPU-1 (1.32).^32^ Isosteric heat of adsorption (*Q*_st_) was calculated by fitting adsorption isotherms at 273, 283, and 298 K using the dual-site Langmuir–Freundlich model (Fig. S10–S19†). At 298 K, the *Q*_st_ of JNU-6 at zero loading was determined to be 24.0 kJ mol^−1^and 20.9 kJ mol^−1^ for C_2_H_6_ and C_2_H_4_, respectively, while the *Q*_st_ of JNU-6-CH_3_ at zero loading was determined to be 24.7 kJ mol^−1^*vs.* 23.9 kJ mol^−1^ for C_2_H_6_ and C_2_H_4_, respectively. The data confirm the stronger thermodynamic affinity toward C_2_H_6_ than C_2_H_4_ in both materials. Moreover, the reduced pore size in JNU-6-CH_3_ may allow for an increased host–guest interaction between the framework and gas molecules, resulting in adsorption affinity stronger than JNU-6 for both C_2_H_6_ and C_2_H_4_. Meanwhile, the *Q*_st_ values of both JNU-6 and JNU-6-CH_3_ are much lower than those of Fe_2_(O_2_)(dobdc) (67 kJ mol^−1^),^17^ IRMOF-8 (52.5 kJ mol^−1^),^33^ PAF-40-Fe (47.8 kJ mol^−1^),^34^ Zn-atz-ipa (45.8 kJ mol^−1^),^35^ and MAF-49 (60 kJ mol^−1^).^12^ The relatively low *Q*_st_ value may facilitate easy regeneration and low energy consumption during the desorption process, reflecting the advantages of pore surface engineering with nonpolar functional groups. Furthermore, ten continuous adsorptions for C_2_H_6_ and C_2_H_4_ were carried out on an ASAP2020 gas sorption instrument. As shown in Fig. S20–S23,† both JNU-6 and JNU-6-CH_3_ retain adsorption capacity over ten cycles, indicating that the samples can be fully regenerated by vacuum at room temperature.

To test their water stability, JNU-6 and JNU-6-CH_3_ were soaked in water for days and then subjected to PXRD and gas adsorption measurements. As shown in Fig. 2c, JNU-6 lost most of the crystallinity and porosity after being soaked in water for three days. In contrast, the crystallinity and structural integrity of JNU-6-CH_3_ can be well retained after being soaked in water for six months (Fig. 2d). Water vapor adsorption measurements for JNU-6 and JNU-6-CH_3_ were carried out and both of them show S-shaped adsorption isotherms characteristic of pore filling (Fig. 4b), and the limited water uptake at low pressure suggests that the water affinity on the MOF surface is relatively low. With the linker methylation, higher water vapor pressure is required to induce the pore filling, indicating further increased hydrophobicity of MOF pores from JNU-6 and JNU-6-CH_3_. Overall, the introduction of CH_3_ groups renders JNU-6-CH_3_ with an optimized pore size, increased surface dipole, and improved hydrolytic stability, which prompted us to further study its potential for C_2_H_6_/C_2_H_4_ separation under humid conditions.

To verify the preferential adsorption of C_2_H_6_ over C_2_H_4_ on JNU-6 and JNU-6-CH_3_, we first performed computational modeling studies using grand canonical Monte Carlo (GCMC) simulations.^35,36^ The simulated C_2_H_6_ and C_2_H_4_ adsorption isotherms are in good agreement with the experimental ones at 298 K and 1 bar, and the probability density distributions of C_2_H_6_ and C_2_H_4_ reveal that both C_2_H_6_ and C_2_H_4_ are preferentially adsorbed at the corners of the cubic-shaped cages in both JNU-6 and JNU-6-CH_3_ (Fig. S24–S27†). Take JNU-6-CH_3_ as an example, there are six C–H⋯π interactions between the H atoms of C_2_H_6_ and the pyrazole rings of the linkers with H⋯π distances from 2.93 to 3.41 Å. In comparison, there are fewer C–H⋯π interactions between C_2_H_4_ and the pyrazole rings of the linkers with H⋯π distances from 3.0 to 3.85 Å (Fig. 3b and e). Further, an independent gradient model based on Hirshfeld partition (IGMH) analysis on the optimized structures was developed. As shown in Fig. 3c and f, multiple green isosurfaces were observed for both C_2_H_6_ and C_2_H_4_, indicating the existence of vdW interactions between the gas molecules and the three pyrazole rings. The static binding energies (Δ*E*) for C_2_H_6_ on JNU-6 and JNU-6-CH_3_ were calculated to be 18.04 and 22.23 kJ mol^−1^, respectively, higher than those for C_2_H_4_ (17.22 and 20.15 kJ mol^−1^). These values further confirm the weak vdW nature of the host–guest interactions between gas molecules and the nonpolar pore surfaces, which favors the adsorption of C_2_H_6_ over C_2_H_4_.

**Fig. 3:**
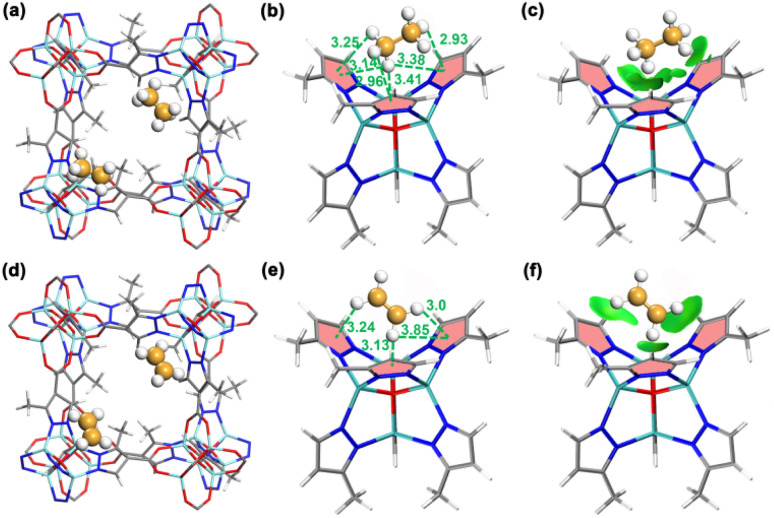
Primary adsorption sites for C_2_H_6_ (a) and C_2_H_4_ (d) in JNU-6-CH_3_ determined by Monte Carlo (GCMC) simulations. C–H⋯π interactions (green dashed lines) for C_2_H_6_ (b) and C_2_H_4_ (e) at the primary adsorption site of JNU-6-CH_3_. Independent gradient model based on Hirshfeld partition (IGMH) analysis for C_2_H_6_ (c) and C_2_H_4_ (f) at the primary adsorption site of JNU-6-CH_3_ (green surfaces represent vdW interactions). (Color code: Zn, cyan; C, dark gray; N, blue; O, red; H, white. The distance unit is in Å).

To evaluate the actual separation capability of JNU-6 and JNU-6-CH_3_ for C_2_H_6_/C_2_H_4_ mixtures, we first performed dynamic column breakthrough experiments in which a C_2_H_6_/C_2_H_4_ (50/50, v/v) mixture was introduced over the activated JNU-6 or JNU-6-CH_3_ at a flow rate of 2 mL min^−1^ and 298 K. As shown in Fig. 4c, JNU-6 can separate C_2_H_6_ from the C_2_H_6_/C_2_H_4_ mixture with an estimated productivity of 4.92 L kg^−1^ of high-purity C_2_H_4_ (≥99.95%) under dry conditions. Surprisingly, JNU-6-CH_3_ exhibits significantly improved separation capacity under similar conditions, and the data are in good agreement with the simulated breakthrough curve (Fig. S29†). As shown in Fig. 4d, C_2_H_4_ and C_2_H_6_ were detected to break through the column at the time points of 52.7 min g^−1^and 67.9 min g^−1^, respectively. During the above time period, high-purity C_2_H_4_ (≥99.95%) can be collected with an estimated C_2_H_4_ productivity of 22.06 L kg^−1^, which is about four times that of JNU-6 and the highest among those of the reported MOFs under similar conditions, including JNU-2 (21.2 L kg^−1^),^13^ Fe_2_(O_2_)(dobdc) (17.7 L kg^−1^),^17^ Tb-MOF-76-(NH_2_) (17.66 L kg^−1^),^37^ TJT-100 (16.38 L kg^−1^),^38^ MUF-15 (14 L kg^−1^),^28^ UiO-67-(NH_2_)_2_ (12.32 L kg^−1^),^5^ MAF-49 (6.23 L kg^−1^),^12^ and Cu(Qc)_2_ (4.0 L kg^−1^)^27^ (Fig. 4f).

**Fig. 4:**
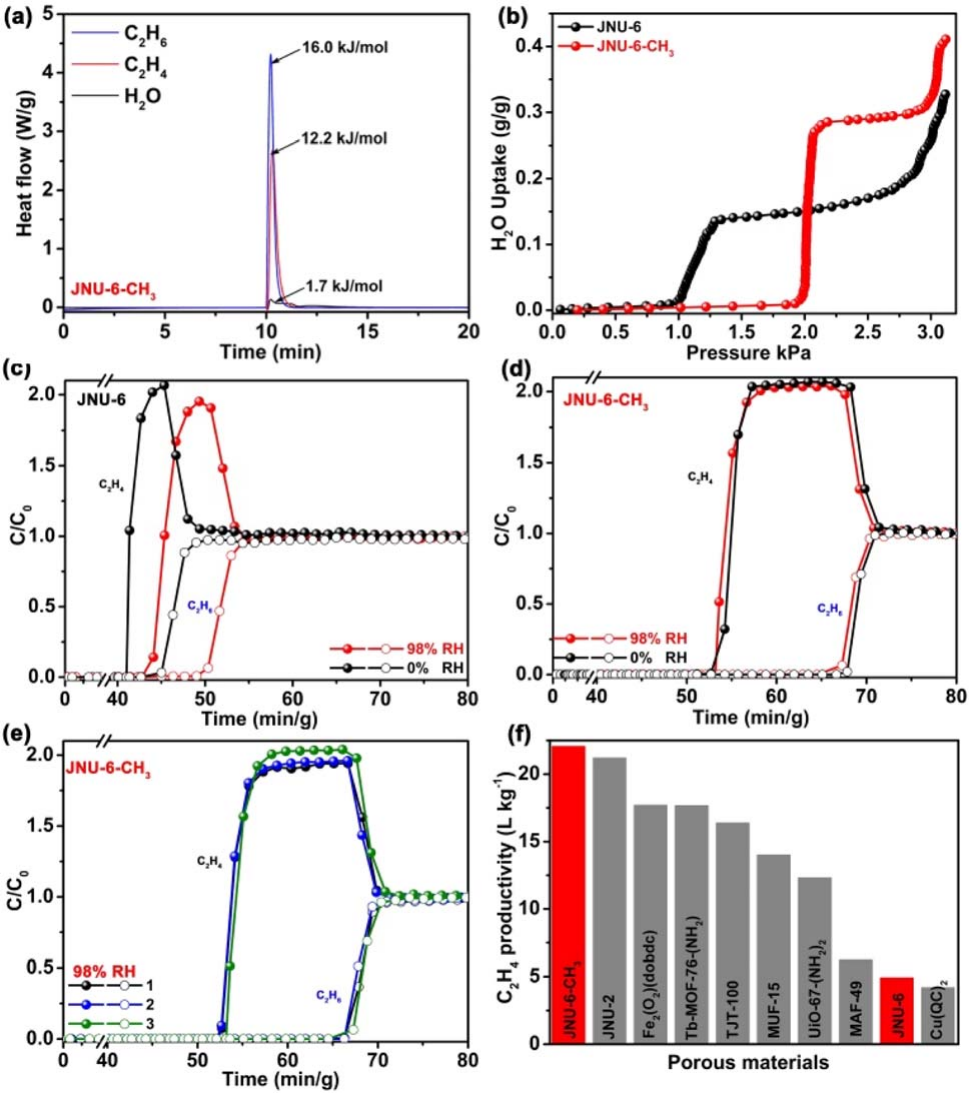
(a) Differential scanning calorimetry (DSC) measurements of heat flow upon introducing C_2_H_6_, C_2_H_4_, and water vapor on JNU-6-CH_3_ at a flow rate of 10 mL min^−1^ at 298 K. (b) Water vapor adsorption isotherms of JNU-6 and JNU-6-CH_3_ at 298 K. (c) Experimental breakthrough curves on JNU-6 (1.0 g) for a C_2_H_6_/C_2_H_4_ (50/50, v/v) mixture at a flow rate of 2.0 mL min^−1^ and 298 K under 0% RH and 98% RH conditions. (d) Experimental breakthrough curves on JNU-6-CH_3_ (0.85 g) for a C_2_H_6_/C_2_H_4_ (50/50, v/v) mixture at a flow rate of 2.0 mL min^−1^ and 298 K under 0% RH and 98% RH conditions. (e) Three cycles of breakthrough experiments on JNU-6-CH_3_ for a C_2_H_6_/C_2_H_4_ (50/50, v/v) mixture at a flow rate of 2.0 mL min^−1^ and 298 K under 98% RH conditions. (f) Comparison of the C_2_H_4_ productivity estimated from breakthrough curves for JNU-6-CH_3_, JNU-6, and other reported porous materials.

To further examine the moisture effect on the separation capability for C_2_H_6_/C_2_H_4_, we performed differential scanning calorimetry (DSC) measurements of heat flow upon introducing water vapor, C_2_H_4_, and C_2_H_6_ on JNU-6 and JNU-6-CH_3_. For JNU-6, the experimental *Q*_st_ for water vapor, C_2_H_4_, and C_2_H_6_ is 0.2, 10.2, and 15.7 kJ mol^−1^, respectively (Fig. S38†), while for JNU-6-CH_3_, the experimental *Q*_st_ for water vapor, C_2_H_4_, and C_2_H_6_ is 1.7, 12.2, and 16.0 kJ mol^−1^, respectively (Fig. 4a), both indicative of significantly stronger binding affinity for C_2_H_6_ and C_2_H_4_ than for water vapor. This, together with water vapor adsorption measurements (Fig. 4b), suggests that JNU-6-CH_3_ may be able to maintain the high separation capability for C_2_H_6_/C_2_H_4_ mixtures under humid conditions. Breakthrough experiments were thus performed on JNU-6 and JNU-6-CH_3_ for a C_2_H_6_/C_2_H_4_ (50/50, v/v) mixture under 98% RH conditions. As revealed in Fig. 4c and d, the purity of C_2_H_4_ dropped from 99.95% to 99.2% for JNU-6, likely due to its hydrolytic instability (Fig. 2c), whereas the purity of C_2_H_4_ remained over 99.95% with only slightly dropped productivity (18.71 L kg^−1^) for JNU-6-CH_3_. The results confirm that the introduction of CH_3_ groups in the framework can indeed improve separation capability, especially under humid conditions. Furthermore, continuous breakthrough experiments under humid conditions were carried out, revealing the retained separation performance of JNU-6-CH_3_ over three cycles (Fig. 4e and S31†).

To further study the effect of methylation degree on adsorption separation performance, we synthesized JNU-6-(CH_3_)_2_ with 3,5-dimethylpyrazole-4-carboxylic acid. JNU-6-(CH_3_)_2_ also shows preferential adsorption of C_2_H_6_ over C_2_H_4_, especially in the low-pressure range (Fig. S33†). However, its C_2_H_6_ and C_2_H_4_ adsorption amounts at 0.5 bar are almost the same, and the adsorption of C_2_H_6_ and C_2_H_4_ on JNU-6-(CH_3_)_2_ is significantly lower than those on JNU-6-CH_3_ and JNU-6 in the high-pressure range, likely due to the reduced porosity of JNU-6-(CH_3_)_2_ (Fig. S32a†). As a result, dynamic column breakthrough experiments on JNU-6-(CH_3_)_2_ reveal a poor separation for a C_2_H_6_/C_2_H_4_ (50/50, v/v) mixture at a flow rate of 2.0 mL min^−1^ and 298 K (Fig. S32d†). On the other hand, JNU-6-CF_3_ was synthesized by using 5-trifluoromethyl-4-carboxylic acid as a ligand. JNU-6-CF_3_ also shows preferential adsorption of C_2_H_6_ over C_2_H_4_. The maximum uptake of C_2_H_6_ on JNU-6-CF_3_ is 3.49 mmol g^−1^ (Fig. S34b†), which is nearly 25% less than that of JNU-6-CH_3_, likely due to the reduced porosity of JNU-6-CF_3_ (Fig. S35a†). The water vapor adsorption isotherm of JNU-6-CF_3_ displays almost no water uptake over the entire pressure range (Fig. S34c†), reflecting its extremely high hydrophobicity. We evaluated dynamic column breakthrough experiments on JNU-6-CF_3_ for a C_2_H_6_/C_2_H_4_ (50/50, v/v) mixture at a flow rate of 2.0 mL min^−1^ and 298 K. As shown in Fig. S34d,† a clean separation of C_2_H_6_ from the C_2_H_6_/C_2_H_4_ mixture can be realized under either dry or 98% RH conditions with no obvious decrease in separation performance. Based on the breakthrough curves, *ca.* 10.5 L kg^−1^ of high-purity C_2_H_4_ (≥99.95%) can be recovered from the C_2_H_4_/C_2_H_6_ (50/50) mixture in a single breakthrough operation, which is about half of that of JNU-6-CH_3_. The results indicate that further increase of methylation degree or introducing more hydrophobic CF_3_ groups may not be necessarily favorable for the C_2_H_6_/C_2_H_4_ separation, and both adsorption capacity and adsorption selectivity have to be considered to achieve high separation efficiency.

In summary, we have successfully demonstrated a surface engineering strategy to boost the separation potential of C_2_H_4_ from C_2_H_6_/C_2_H_4_ mixtures under either dry or humid conditions. The introduction of CH_3_ groups on an isoreticular MOF-5 analogue (JNU-6) renders the obtained JNU-6-CH_3_ with enhanced hydrolytic stability and a more suitable pore environment for C_2_H_6_/C_2_H_4_ separation. JNU-6-CH_3_ retains its framework integrity even after being immersed in water for six months, and it exhibits large C_2_H_6_ adsorption capacity (4.63 mmol g^−1^) and high C_2_H_6_/C_2_H_4_ adsorption selectivity (1.67) due to the optimized pore size and surface function. Breakthrough experiments reveal benchmark productivity of 22.06 and 18.71 L kg^−1^ of high-purity C_2_H_4_ (≥99.95%) from a C_2_H_6_/C_2_H_4_ (50/50, v/v) mixture under dry and humid conditions, respectively. This work offers a promising approach for designing MOFs to overcome the adsorption/selectivity trade-off limitation in paraffin/olefin separation.

## Data availability

The data that support the plots within this paper and other findings of this study are available from the corresponding authors upon reasonable request. The X-ray crystallographic coordinates for structures reported in this Article have been deposited at the Cambridge Crystallographic Data Centre (CCDC), under deposition numbers CCDC 2259108, 2258075, and 2286047.†https://www.ccdc.cam.ac.uk/data_request/cif

## Author contributions

H. Z., W. L., and D. L. conceived and designed the research. X.-J. X., H. Z., and W. L. co-wrote the manuscript. X.-J. X. and Q.-Y. C. planned and executed the synthesis, characterization, and gas separation studies. Y. W. and R. H. performed the theoretical simulations. X.-J. X. carried out the structural analyses. All authors participated in and contributed to the preparation of the manuscript.

## Conflicts of interest

There are no conflicts to declare.

## Supplementary Material

SC-014-D3SC04119K-s001

SC-014-D3SC04119K-s002
