# Supplementary material for: Surface engineering on a microporous metal–organic framework to boost ethane/ethylene separation under humid conditions
Source: Chem Sci. 2023 Oct 9;14(42):11890–5. doi: 10.1039/d3sc04119k (PMC10619615; doi:10.1039/d3sc04119k)
Supplement: SC-014-D3SC04119K-s001 [file SC-014-D3SC04119K-s001.pdf]

Electronic Supplementary Material (ESI) for Chemical Science.
This journal is © The Royal Society of Chemistry 2023

Supporting Information

Surface Engineering on a Microporous Metal-Organic Framework to Boost Ethane/Ethylene Separation under Humid Conditions

Xiao-Jing Xie,[a] Ying Wang,[a] Qi-Yun Cao,[a] Rajamani Krishna,[b] Heng Zeng,[*a] Weigang Lu,[*a] Dan Li[a]

[a]College of Chemistry and Materials Science, Guangdong Provincial Key Laboratory of Functional Supramolecular Coordination Materials and Applications, Jinan University, Guangzhou 510632, P. R. China.

[b]Van't Hoff Institute for Molecular Sciences, University of Amsterdam, Science Park 904, Amsterdam 1098 XH, Netherlands.

[*]Corresponding author. Email: zengheng90@163.com; weiganglu@jnu.edu.cn;

Materials and synthesis methods

Materials

All reagents and materials were commercially available and used as received without further purification.

Syntheses of JNU-6

A mixture of pyrazole-4-carboxylic acid (300 mg, 2.68 mmol), $Zn(NO_3)_2{\cdot}6H_2O$ (7140 mg, 24 mmol), and N,N-Diethylformamide (DEF, 120 mL) was placed in a 350 mL of glass vial and heated at 100 °C for 12 h. After cooling down to room temperature, the block crystals were washed with methanol 10 times at 25 °C and then dried under high vacuum at 90 °C.

Syntheses of JNU-6-CH_3 and JNU-6-$(CH_3)_2$

A mixture of 3-methylpyrazole-4-carboxylic acid (378 mg, 3.25 mmol), $Zn(NO_3)_2{\cdot}6H_2O$ (900 mg, 3.02 mmol), N,N-Diethylformamide (DEF, 120 mL), deionized water (30 mL), and nitric acid (0.1 mol/L, 4.5 mL) was placed in a 350 mL of glass vial and heated at 120 °C for 12 h. After cooling down to room temperature, the block crystals were washed with methanol 10 times at 70 °C and then dried under high vacuum at 200 °C. The syntheses of JNU-6-$(CH_3)_2$ is consistent with that of JNU-6-CH_3.

Syntheses of JNU-6-CF_3

A mixture of 5-trifluoromethyl-4-carboxylic acid (560 mg, 4.0 mmol), $Zn(NO_3)_2{\cdot}6H_2O$ (1200 mg, 4.02 mmol), *N,N*-Dimethylformamide (DMF, 90 mL), ethanol (30 mL) was placed in a 350 mL of glass vial and heated at 120 °C for 12 h. After cooling down to room temperature, the block crystals were washed with methanol 10 times at 70 °C and then dried under high vacuum at 200 °C.

Gas adsorption measurement

At least 100 mg of sample were activated under dynamic vacuum (below 5 μmHg) for 24 h. Single-component gas adsorption isotherms were obtained on an ASAP 2020 PLUS Analyzer (Micromeritics).

Powder X-ray diffraction (PXRD) analysis

Powder X-ray diffraction data were recorded with microcrystalline samples on a Rigaku Ultima IV diffractometer (40 kV, 40 mA, Cu Kα, λ = 1.5418 Å). The measurement parameters include a scan speed of 10°/min, a step size of 0.02°, and a scan range of 2θ from 5° to 30°. For variable temperature PXRD measurements, the measurement parameters include a scan speed of 2°/min, a step size of 0.02°, and a scan range of 2θ from 5° to 30°.

Thermogravimetric analysis (TGA)

About 5 mg of dried samples was used on a Q50 thermogravimetric analyzer (TGA) from 40 to 800 °C under a N_2 flow with a heating rate of 10 °C/min.

The isosteric enthalpy of adsorption (Q_{st})

The unary isotherm data for C_2H_6 and C_2H_4, measured at three different temperatures 273 K, 283 K, and 298 K in JNU-6 and JNU-6-CH_3 were fitted with excellent accuracy using the dual-site Langmuir-Freundlich model, where we distinguish two distinct adsorption sites A and B:

$$q = q_{sat,A} \frac{b_A p^{vA}}{1+b_A p^{vA}} + q_{sat,B} \frac{b_B p^{vB}}{1+b_B p^{vB}} \qquad \text{(S1)}$$

In eq S1, the Langmuir-Freundlich parameters b_A and b_B are both temperature dependent

$$b_A = b_{A0} \exp\left(\frac{E_A}{RT}\right);\ b_B = b_{B0} \exp\left(\frac{E_B}{RT}\right) \qquad \text{(S2)}$$

In eq S2, E_A, E_B are the energy parameters associated with sites A, and B, respectively.

The fit parameters are provided in Table S2, and Table S3.

The isosteric heat of adsorption, Q_{st}, is defined as

$$Q_{st} = -RT^2 \left(\frac{\partial \ln p}{\partial T}\right)_q \qquad \text{(S3)}$$

where the derivative in the right member of eq S3 is determined at constant adsorbate loading, q. The derivative was determined by analytic differentiation of the combination of eq S1, eq S2, and eq S3.

IAST selectivities and separation potential

A key metric that quantifies the efficacy of a MOF for separation of binary C_2H_6(1)/C_2H_4(2) mixtures is the adsorption selectivity, S_{ads}, defined by

$$S_{ads} = \frac{q_1/q_2}{p_1/p_2} \tag{S1}$$

where q_1 and q_2 are the molar loadings of the guest components in the adsorbed phase in equilibrium with a bulk gas phase mixture with partial pressures p_1 and p_2. The mixture adsorption equilibrium is commonly determined using the Ideal Adsorbed Solution theory (IAST)[1] using fits of unary isotherms as input data.

These mixture separations are envisaged to be carried out in fixed bed adsorbers. In such devices, the separations are dictated by a combination of adsorption selectivity and uptake capacity. Using the shock wave model for fixed bed adsorbers, Krishna[2, 3] has suggested that the appropriate metric is the separation potential, Δq_1.

$$\Delta q = q_1 \frac{y_{20}}{y_{10}} - q_2 \tag{S5}$$

In eq S5 y_{10}, y_{20} are the mole fractions of the feed mixture during the adsorption cycle. In the derivation of eq S5, it is assumed that the concentration "fronts" traversed the column in the form of shock waves during the desorption cycle. The molar loadings q_1, q_2 of the two components are determined using the Ideal Adsorbed Solution Theory (IAST) of Myers and Prausnitz using the unary isotherm fits as data inputs.[1] The physical significance of Δq is the maximum productivity of pure C_2H_4 (2) that is achievable in the adsorption cycle of PSA operations.

The IAST calculations of S_{ads}, and Δq were performed for binary 50/50 C_2H_6(1)/C_2H_4 (2) mixtures at 298 K, at total pressures ranging from 1 to 100 kPa.

Computational details

Grand Canonical Monte Carlo (GCMC) simulations were performed to simulate the single-component adsorption of C_2H_6 and C_2H_4 on JNU-6 and JNU-6-CH_3 by RASPA2 software.[2,3] These frameworks were considered to be rigid, and the optimized gas molecules were used. The interaction energies between the gas molecules and framework were computed through the Coulomb and Lennard-Jones 6-12 (LJ) potentials. The simulation box of the GCMC run was constructed by 2×2×2 supercell of the respective MOFs, and the cut-off radius was chosen as 14 Å. The Lennard–Jones (LJ) parameters for frameworks were taken from Dreiding force field[4], and if not available, from the universal force field (UFF).[5] The LJ parameters for gas molecules were taken from literature.[6,7] The LJ parameters of different atom types were computed using the Lorentz-Berthelot mixing rules. The long-range electrostatic interactions were calculated by using Ewald summation. The equilibration steps and production steps were both set as 1.0×10^7. The DDEC charges[8] calculated by the

Vienna ab initio simulation package (VASP)[9,10], were employed to the framework atoms. Perdew-Burke-Ernzerhof (PBE) functional with generalized gradient approximation (GGA) was used to evaluate the electron exchange correlation.

To further quantify the binding energies between framework and gas molecules, dispersion-corrected density functional theory (DFT-D) calculations were performed based on the cluster models extracted from the structures of JNU-6 and JNU-6-CH_3. The truncated bonds of the cluster models were saturated with hydrogen atoms or methyl groups. All geometry optimizations were performed at the B3LYP-D3(BJ)/6-31G* level for the non-metal atoms.[11-13] For Zn atom, the LanL2DZ basis set[14] was used to consider the relativistic effects. Frequency analyses were performed at the same computational level to confirm local minima for each optimized structure. Based on the optimized geometries, these binding energies (ΔE) were corrected from the basis set superposition error (BSSE) by the counterpoise procedure.[15] All these DFT-D calculations were accomplished using Gaussian 16 software.[16] The binding energy (ΔE) was calculated by the following equation:

$$\Delta E = E_{gas+MOF} - E_{MOF} - E_{gas} + E_{BSSE}$$

Where $E_{gas+MOF}$, E_{MOF}, E_{gas} are the optimization energy of MOF with an adsorbed gas molecule, MOF structure and isolated gas molecule, respectively. while the E_{BSSE} can correct for weak intermolecular interactions.

To reveal the nature of the intermolecular interaction vividly, the electrostatic potential (ESP) on van der Waals (vdW) surface[17,18] and the independent gradient model based on Hirshfeld partition (IGMH) analyses[19] were performed. The ESP and IGMH analyses were achieved by Multiwfn 3.8 program[20] based on the wave function files generated by DFT-D calculations. Molecular graphs of ESP and IGMH maps were rendered by means of Visual Molecular Dynamics (VMD) 1.9.3 software.[21]

Column breakthrough experiments:

The breakthrough experiments were carried out under ambient conditions (298 K, 1 bar) by using a lab-scale fixed-bed system (Figs. S36 and S37). The activated sample JNU-6 (1.01 g) , JNU-6-CH_3 (0.85 g), JNU-6-$(CH_3)_2$ (0.92 g), and JNU-6-CF_3 (0.9 g) were packed into a custom-made stainless-steel column (3.15 mm ID × 450 mm) and then was activated under high vacuum for 12 h.

For C_2H_6/C_2H_4 and $C_2H_2/C_2H_6/C_2H_4$, the gas mixture of C_2H_6/C_2H_4 (1/1, v/v) or $C_2H_6/C_2H_4/C_2H_2$ (1/1/1, v/v/v) was introduced into breakthrough apparatus with a total flow rate of 2.0 mL·min^{-1}. The outlet effluent of the column was continuously

monitored using a gas chromatograph (GC-7890B, Agilent) with a thermal conductivity detector (TCD).

For $C_2H_6/C_2H_4/CO_2$, the gas mixture of $C_2H_6/C_2H_4/CO_2$ (1/1/1, v/v/v) was introduced into breakthrough apparatus with a total flow rate of 2.0 $mL \cdot min^{-1}$. The outlet effluent of the column was continuously monitored using a gas chromatograph (GC-7890B, Agilent) with a thermal conductivity detector (TCD).

The sample was regenerated *in-situ* in the column at 298 K with helium sweeping for 12 h in the cyclic test. The complete breakthrough of C_2H_6 was indicated by the downstream gas composition reaching that of the feed gas. On the basis of the mass balance, the gas adsorption capacities can be determined as follows[22]:

$$q_i = \frac{C_i V}{22.4 \times m} \times \int_0^t \left(1 - \frac{F}{F_0}\right) dt$$

Where q_i is the equilibrium adsorption capacity of gas i (mmol/g), C_i is the feed gas concentration, V is the volumetric feed flow rate (mL/min), t is the adsorption time (min), F_0 and F are the inlet and outlet gas molar flow rates, respectively, and m is the mass of the adsorbent (g).

The C_2H_6 purity (c) is defined by the peak area of C_2H_6, we calculated C_2H_6 purity according to the following equation:

$$c = \frac{C_i(C_2H_6)}{C_i(C_2H_6) + C_i(C_2H_4)}$$

where C_i (C_2H_6) and C_i (C_2H_4) represent the peak areas of component C_2H_6 and C_2H_4 in a single injection.

Transient breakthrough simulations

Transient breakthrough simulations were carried out for binary C_2H_6/C_2H_4 (50/50) feed mixture at 298 K and 100 kPa total pressure using JNU-6-CH_3. The simulation methodology is described in earlier publications.[23-27] In these simulations, the intra-crystalline diffusional influences are considered to be of negligible importance.

The bed dimensions and operating conditions are the same as in the experiments: length of packed bed, L = 450 mm; inside tube diameter = 3.15 mm; volumetric flow rate of gas mixture at the entrance to the bed, $Q_0 = 2$ mL min^{-1}; mass of JNU-6-CH_3 in packed tube = 0.85 g.

Table S1 Comparison of molecular sizes and physical properties of C_2H_4 and C_2H_6.[28]

**Molecule**	**Boiling point (°C)**	**Polarizability (×10⁻²⁵ cm³)**	**Kinetic diameter (Å)**	**Molecular size (Å³)**
C_2H_4	169.4	42.52	4.163	3.28 × 4.18 × 4.84
C_2H_6	184.5	44.3 – 44.7	4.443	4.08 × 3.81 × 4.82

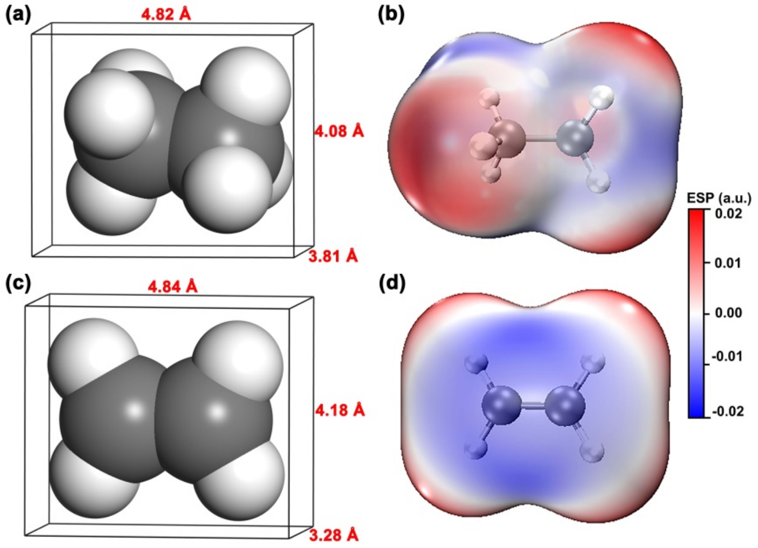

Fig. S1 (a) Molecular size and (b) electrostatic potential of C_2H_6. (c) Molecular size and (d) electrostatic potential of C_2H_4. Electrostatic potential (ESP) analysis was performed by the Multiwfn software package.[29,30]

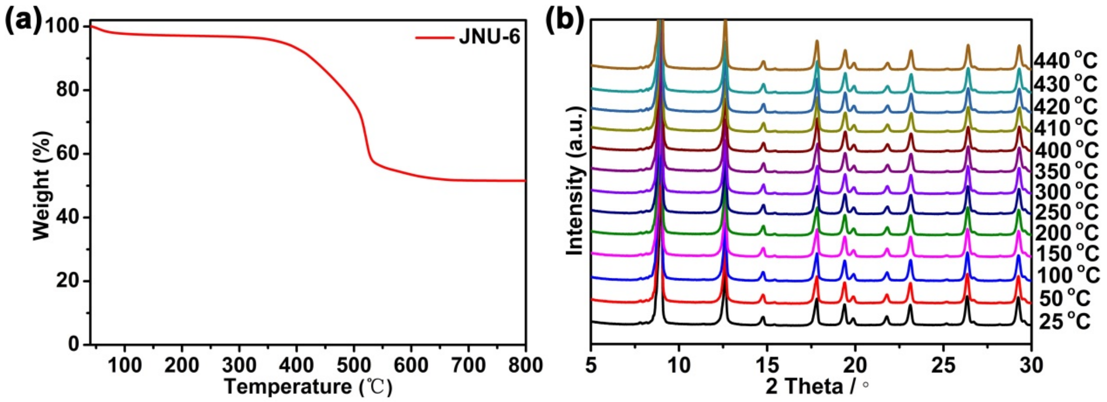

Fig. S2 (a) Thermogravimetric analysis (TGA) curves of the activated JNU-6. (b) *In-situ* variable-temperature PXRD (VT-PXRD) patterns of JNU-6 under the N_2 atmosphere.

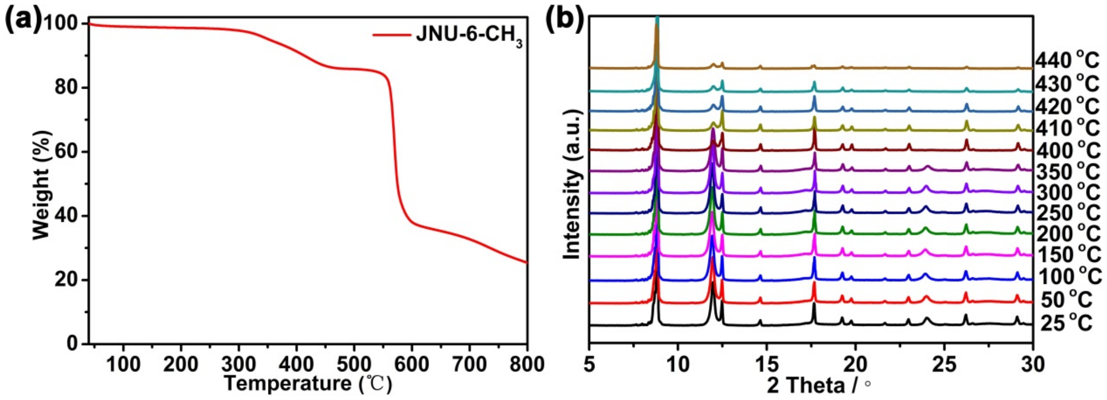

Fig. S3 (a) Thermogravimetric analysis (TGA) curves of the activated JNU-6-CH_3. (b) *In-situ* variable-temperature PXRD (VT-PXRD) patterns of JNU-6-CH_3 under the N_2 atmosphere.

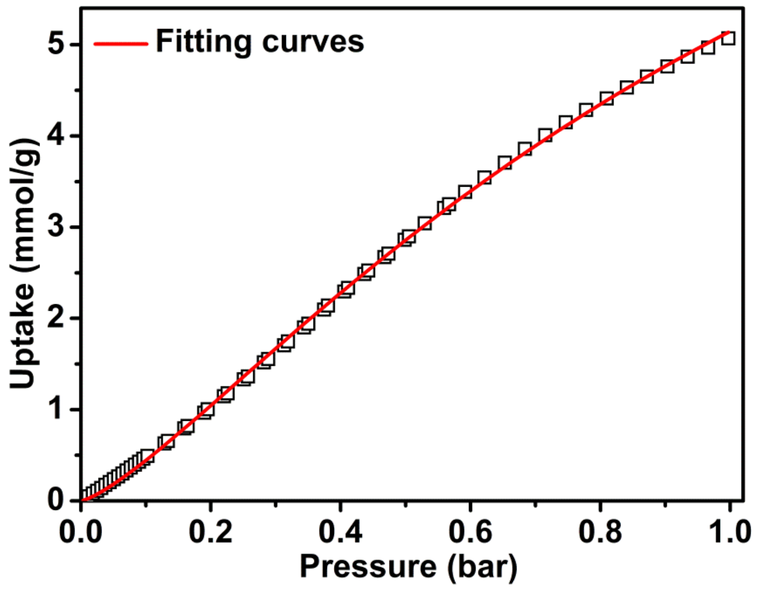

Fig. S4 Dual-site Langmuir-Freundlich fitting for the C_2H_6 adsorption isotherm of JNU-6 at 298 K.

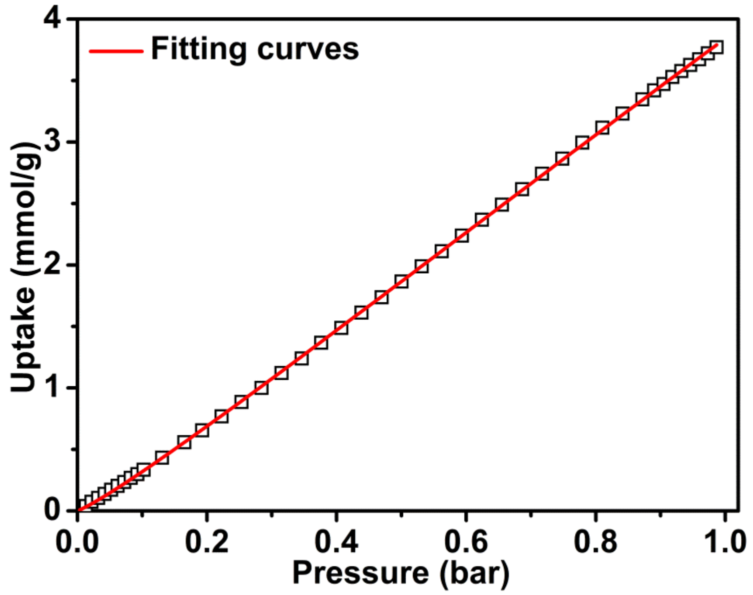

Fig. S5 Dual-site Langmuir-Freundlich fit for the C_2H_4 adsorption isotherm of JNU-6 at 298 K.

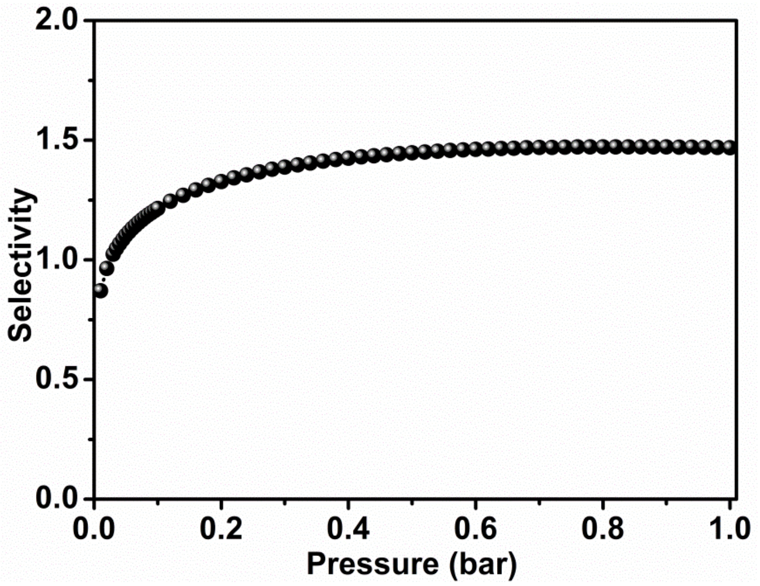

Fig. S6 IAST selectivity of JNU-6 for an equimolar C_2H_6/ C_2H_4 mixture at 298 K.

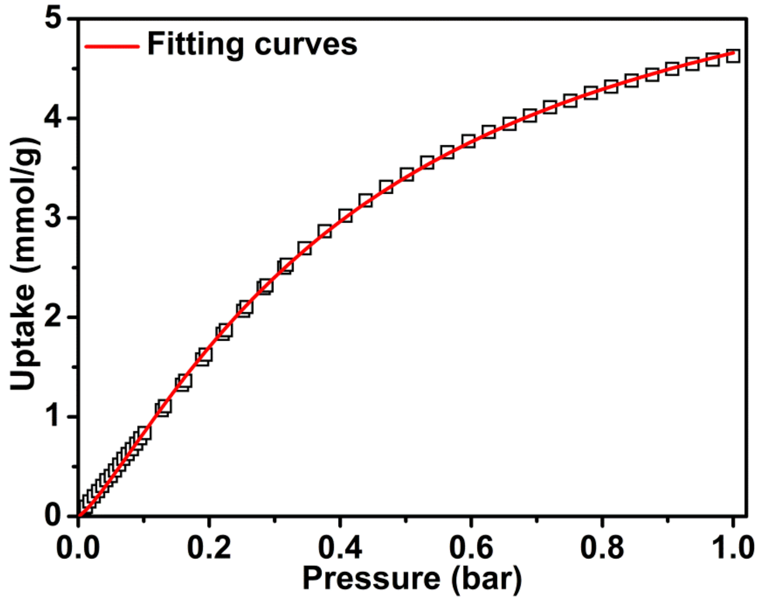

Fig. S7 Dual-site Langmuir-Freundlich fitting for the C_2H_6 adsorption isotherm of JNU-6-CH_3 at 298 K.

Fig. S8 Dual-site Langmuir-Freundlich fitting for the C_2H_4 adsorption isotherm of JNU-6-CH_3 at 298 K.

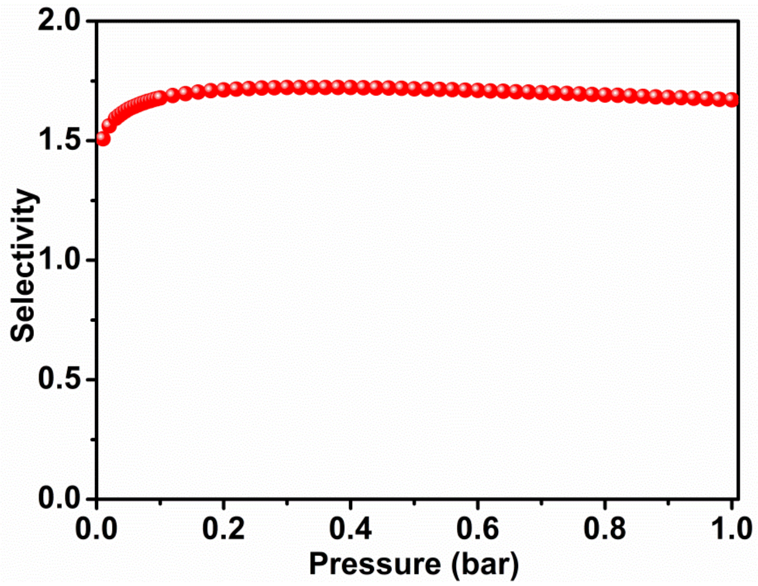

Fig. S9 IAST selectivity of JNU-6-CH_3 for an equimolar C_2H_6/C_2H_4 mixture at 298 K.

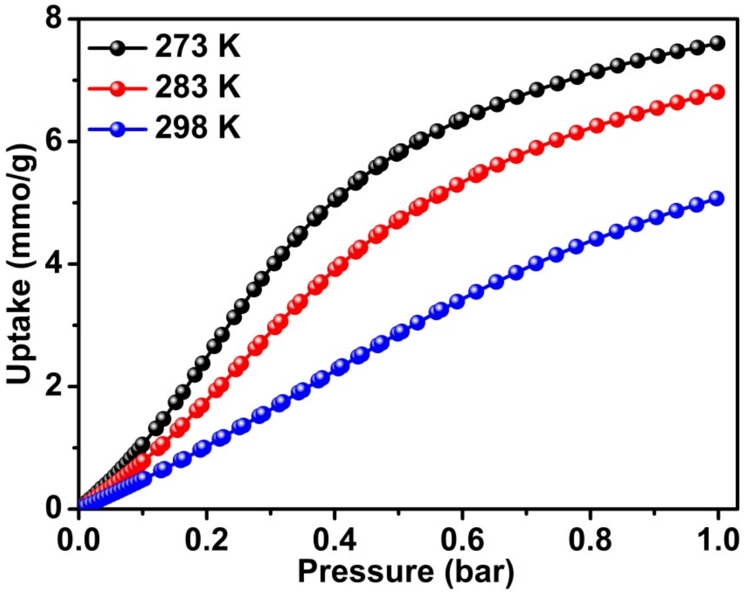

Fig. S10 C_2H_6 adsorption isotherms of JNU-6 at 273, 283, and 298 K.

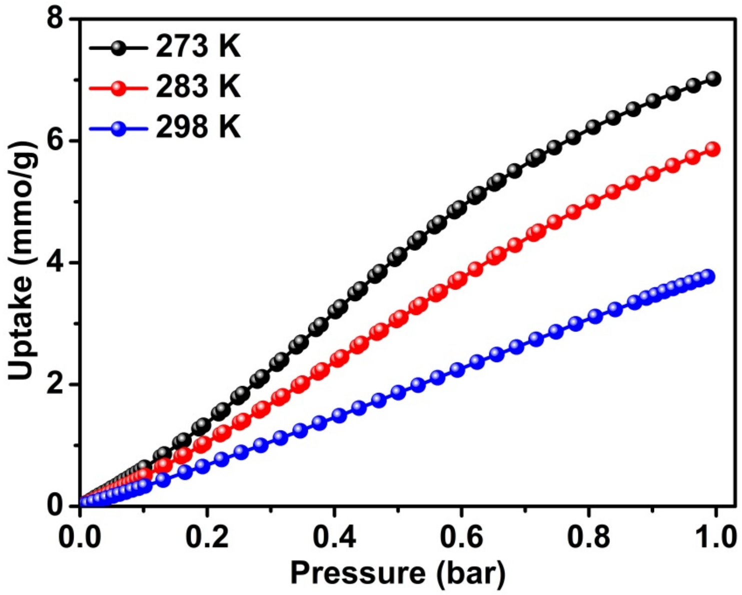

Fig. S11 C_2H_4 adsorption isotherms of JNU-6 at 273, 283, and 298 K.

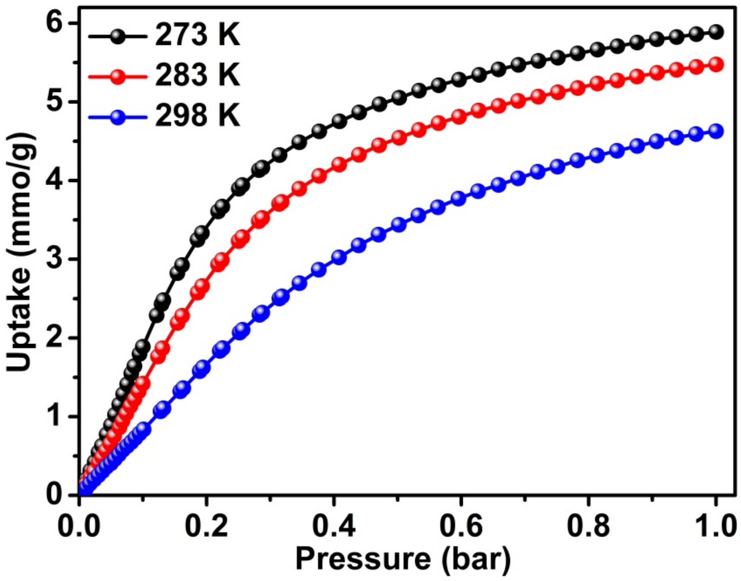

Fig. S12 C_2H_6 adsorption isotherms of JNU-6-CH_3 at 273, 283, and 298 K.

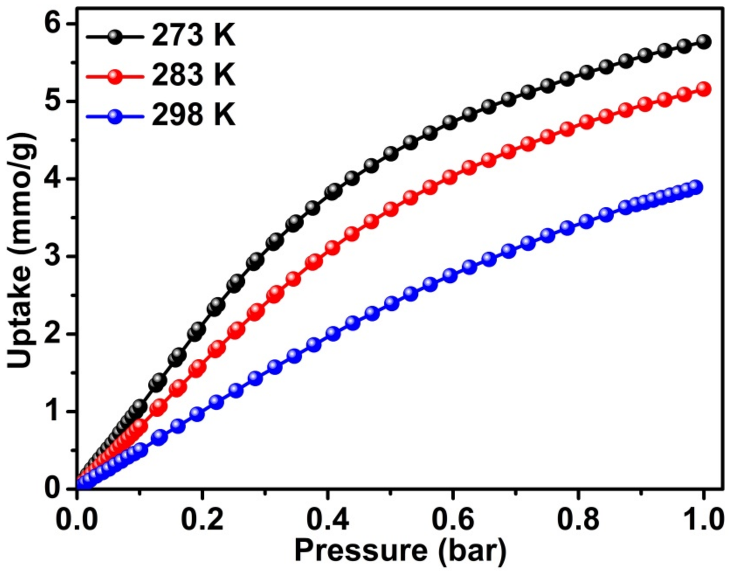

Fig. S13 C_2H_4 adsorption isotherms of JNU-6-CH_3 at 273, 283, and 298 K.

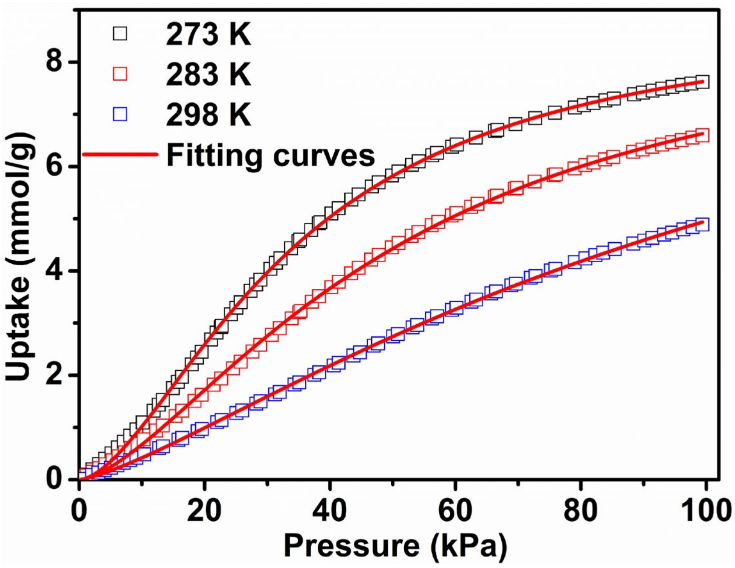

Fig. S14 Dual-site Langmuir-Freundlich fitting of the C_2H_6 adsorption isotherms of JNU-6 at 273, 283, and 298 K.

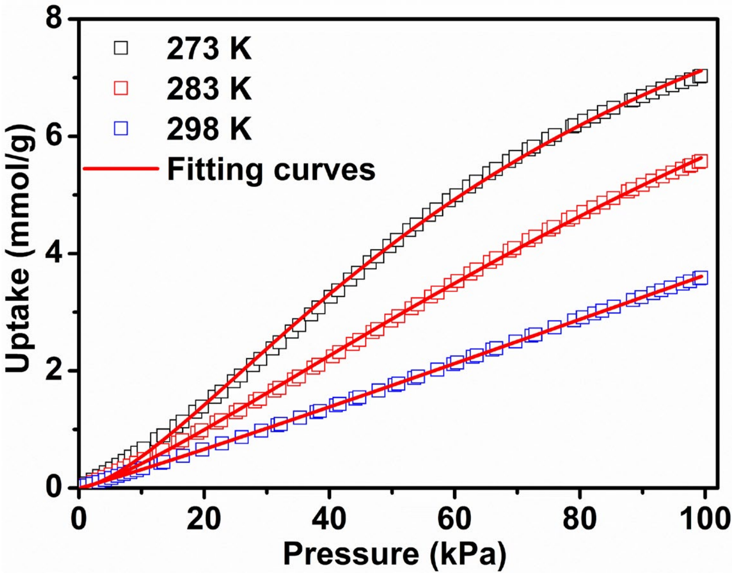

Fig. S15 Dual-site Langmuir-Freundlich fitting of the C_2H_4 adsorption isotherms of JNU-6 at 273, 283, and 298 K.

Table 2. Dual-site Langmuir-Freundlich fits for C_2H_6 and C_2H_4 in JNU-6.

	Site A				Site B			
	$\frac{q_{A,sat}}{\text{mol kg}^{-1}}$	$\frac{b_{A,0}}{\text{Pa}^{-v_A}}$	$\frac{E_A}{\text{kJ mol}^{-1}}$	v_A	$\frac{q_{B,sat}}{\text{mol kg}^{-1}}$	$\frac{b_{B,0}}{\text{Pa}^{-v_B}}$	$\frac{E_B}{\text{kJ mol}^{-1}}$	v_B
C_2H_6	2.55	1.067E-16	15.9	0.88	11.2	1.930E-12	30.3	1.26
C_2H_4	2.55	1.067E-16	16	1	41	3.840E-11	22.7	1.085

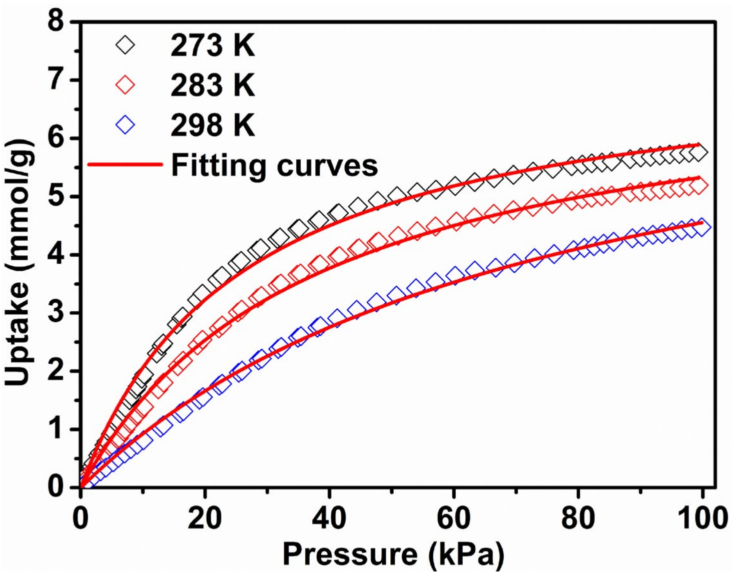

Fig. S16 Dual-site Langmuir-Freundlich fitting of the C_2H_6 adsorption isotherms of JNU-6-CH_3 at 273, 283, and 298 K.

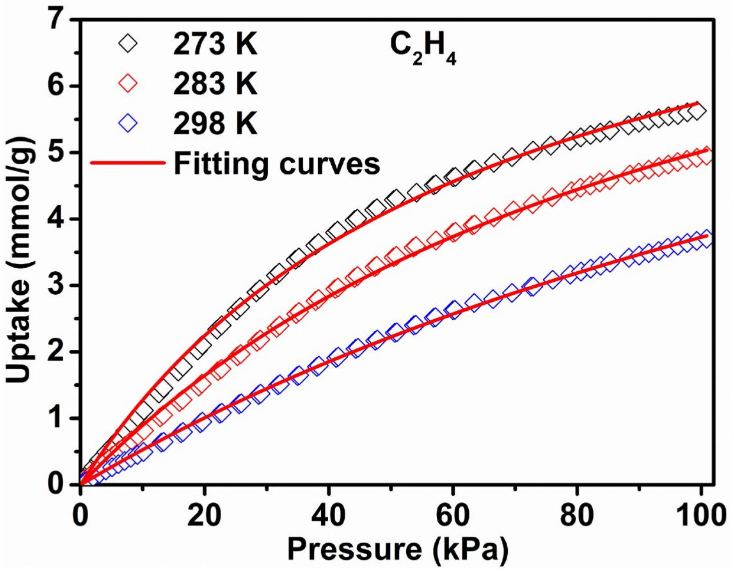

Fig. S17 Dual-site Langmuir-Freundlich fitting of the C_2H_4 adsorption isotherms of JNU-6-CH_3 at 273, 283, and 298 K.

Table S3. Dual-site Langmuir-Freundlich fits for C_2H_6, and C_2H_4 in JNU-6-CH_3.

	Site A				Site B			
	$\frac{q_{A,sat}}{\text{mol kg}^{-1}}$	$\frac{b_{A,0}}{\text{Pa}^{-\nu_A}}$	$\frac{E_A}{\text{kJ mol}^{-1}}$	ν_A	$\frac{q_{B,sat}}{\text{mol kg}^{-1}}$	$\frac{b_{B,0}}{\text{Pa}^{-\nu_B}}$	$\frac{E_B}{\text{kJ mol}^{-1}}$	ν_B
C_2H_6	2.55	1.067E-16	16	1	7.1	1.671E-10	26.5	1.075
C_2H_4	2.55	1.067E-16	16	1	9.7	2.574E-10	24.4	1.02

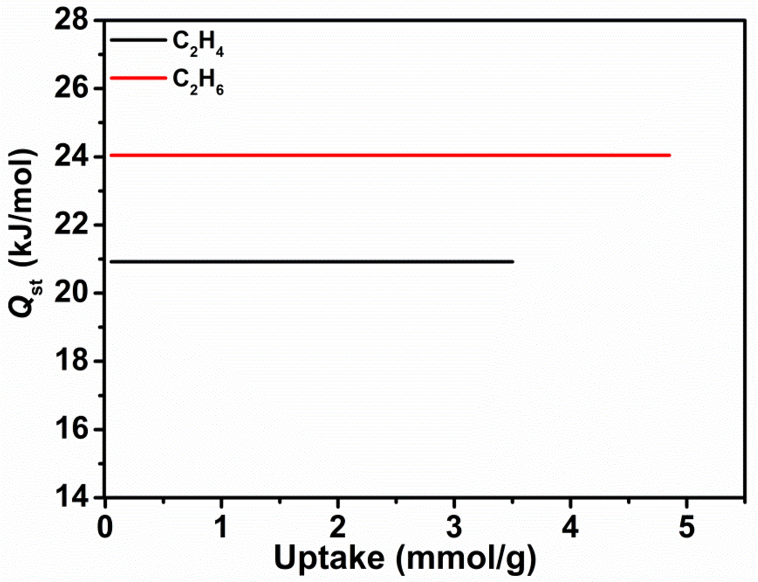

Fig. S18 Calculated C_2H_6 and C_2H_4 adsorption enthalpy (Q_{st}) of JNU-6.

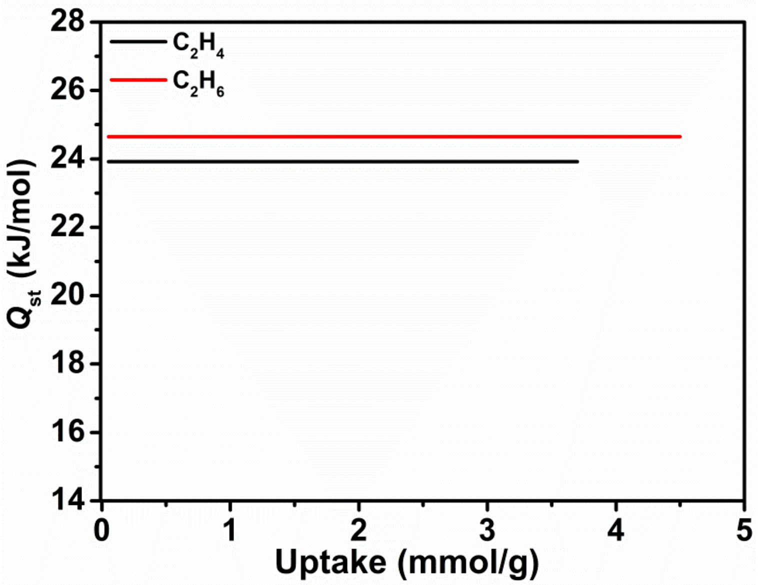

Fig. S19 Calculated C_2H_6 and C_2H_4 adsorption enthalpy (Q_{st}) of JNU-6-CH_3.

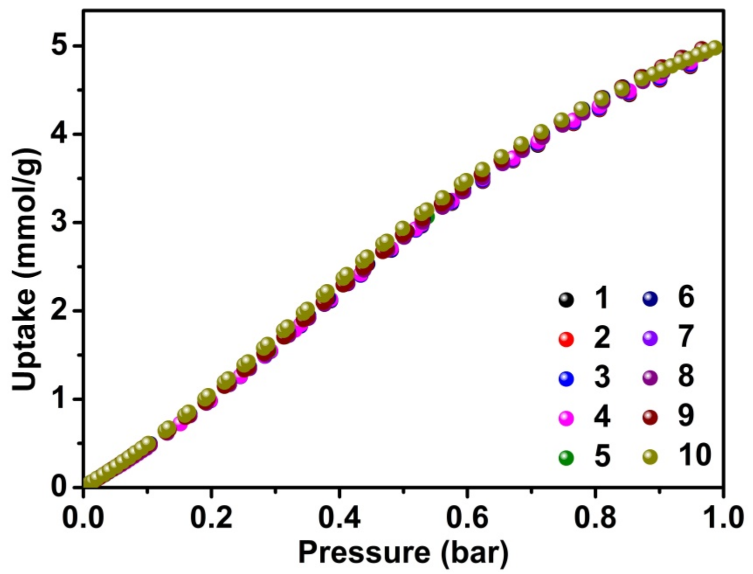

Fig. S20 Continuous C_2H_6 adsorption measurements on JNU-6 at 298 K.

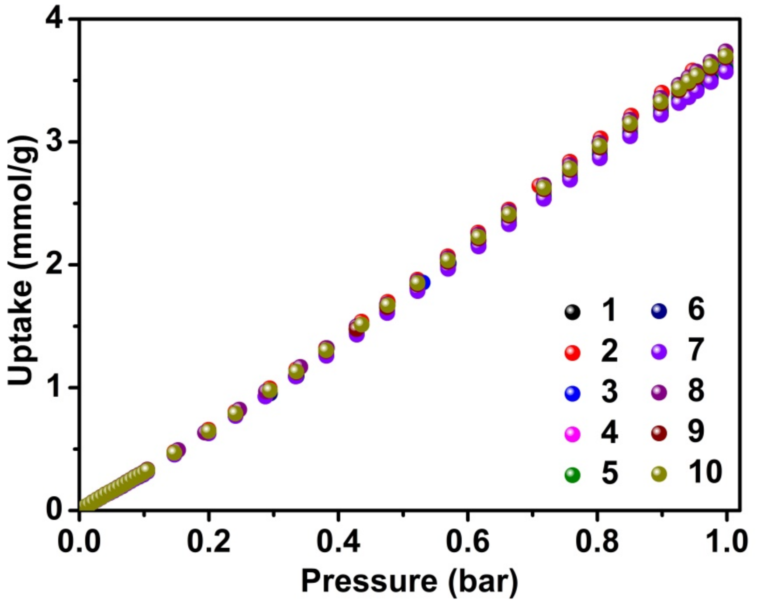

Fig. S21 Continuous C_2H_4 adsorption measurements on JNU-6 at 298 K.

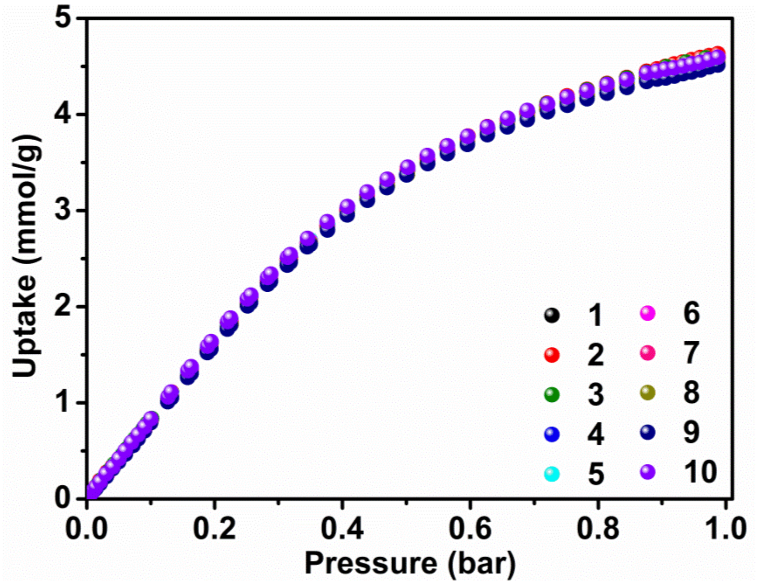

Fig. S22 Continuous C_2H_6 adsorption measurements on JNU-6-CH_3 at 298 K.

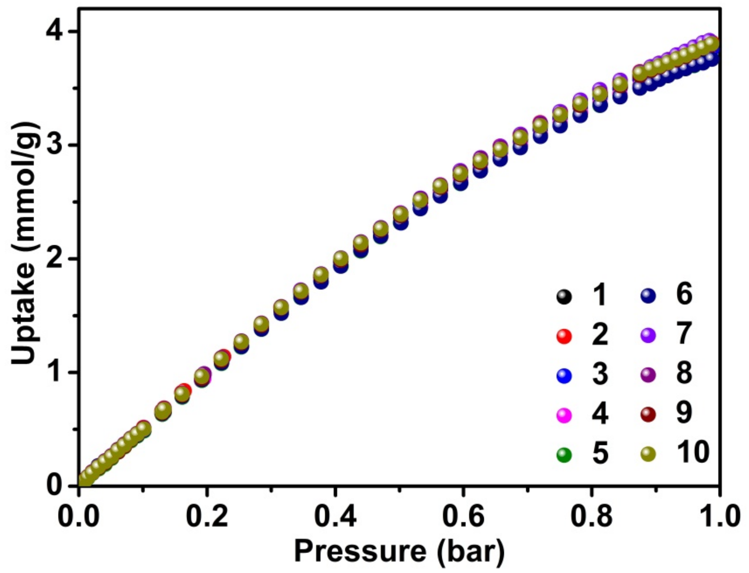

Fig. S23 Continuous C_2H_4 adsorption measurements on JNU-6-CH_3 at 298 K.

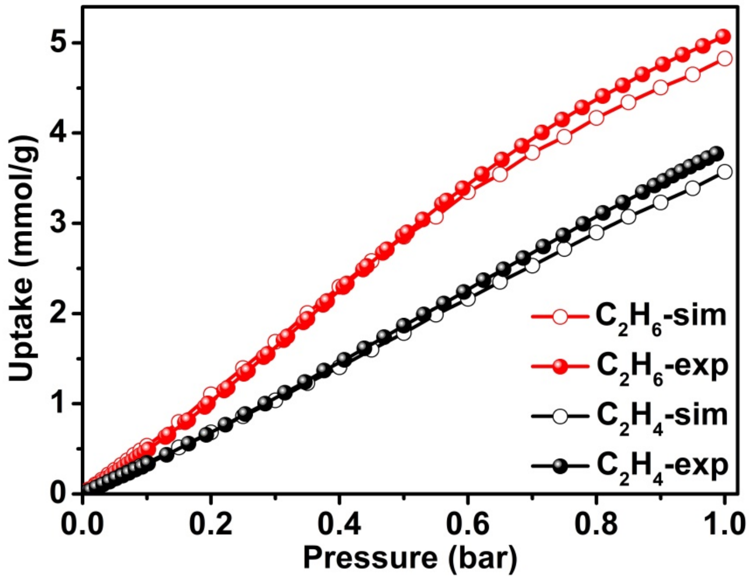

Fig. S24 Experimental and simulated adsorption isotherms of JNU-6 for C_2H_6 (red) and C_2H_4 (black) at 298 K (0-1 bar).

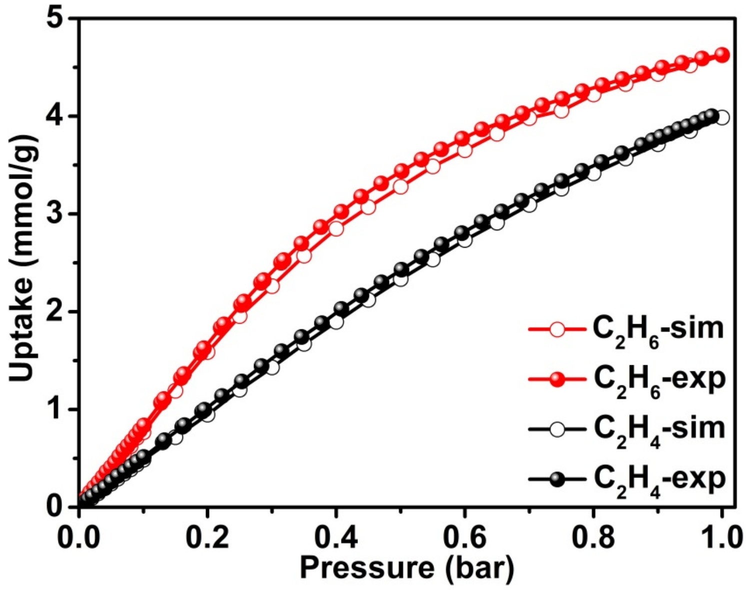

Fig. S25 Experimental and simulated adsorption isotherms of JNU-6-CH_3 for C_2H_6 (red) and C_2H_4 (black) at 298 K.

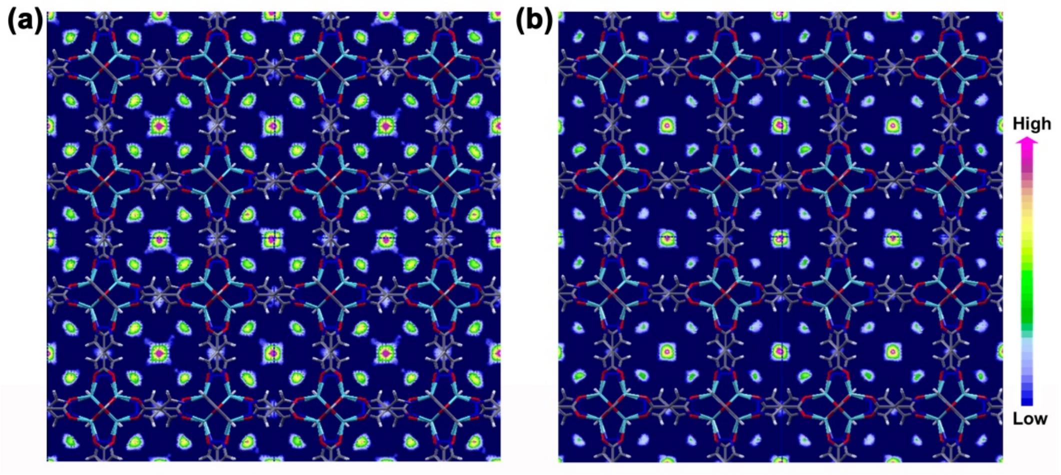

Fig. S26 Contour plots of the COM probability density distributions of (a) C_2H_6 and (b) C_2H_4 for the adsorbed in JNU-6 at 298 K and 1.0 bar. The MOF structure is displayed in a stick style for clarity (atom colors: Zn, cyan; O, red; N, blue; C, gray; H, white).

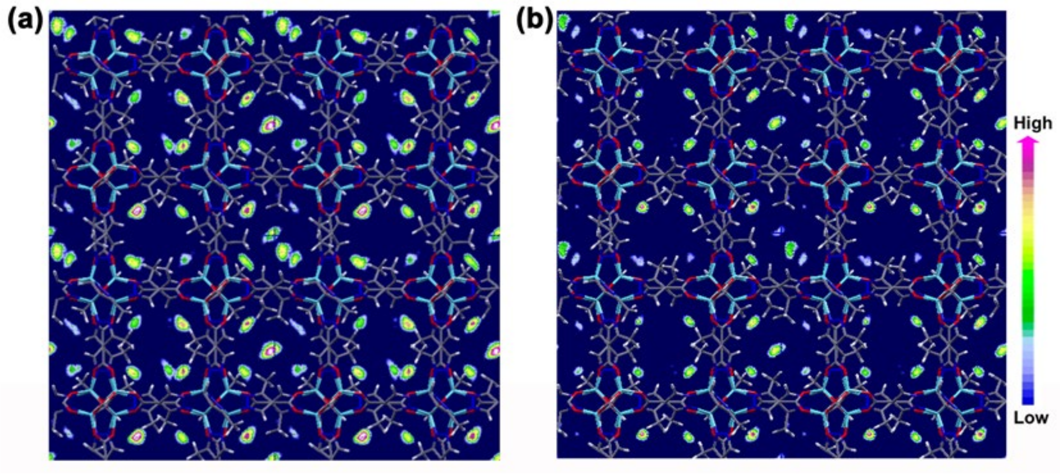

Fig. S27 Contour plots of the COM probability density distributions of (a) C_2H_6 and (b) C_2H_4 for the adsorbed in JNU-6-CH_3 at 298 K and 1.0 bar. The MOF structure is displayed in a stick style for clarity (atom colors: Zn, cyan; O, red; N, blue; C, gray; H, white).

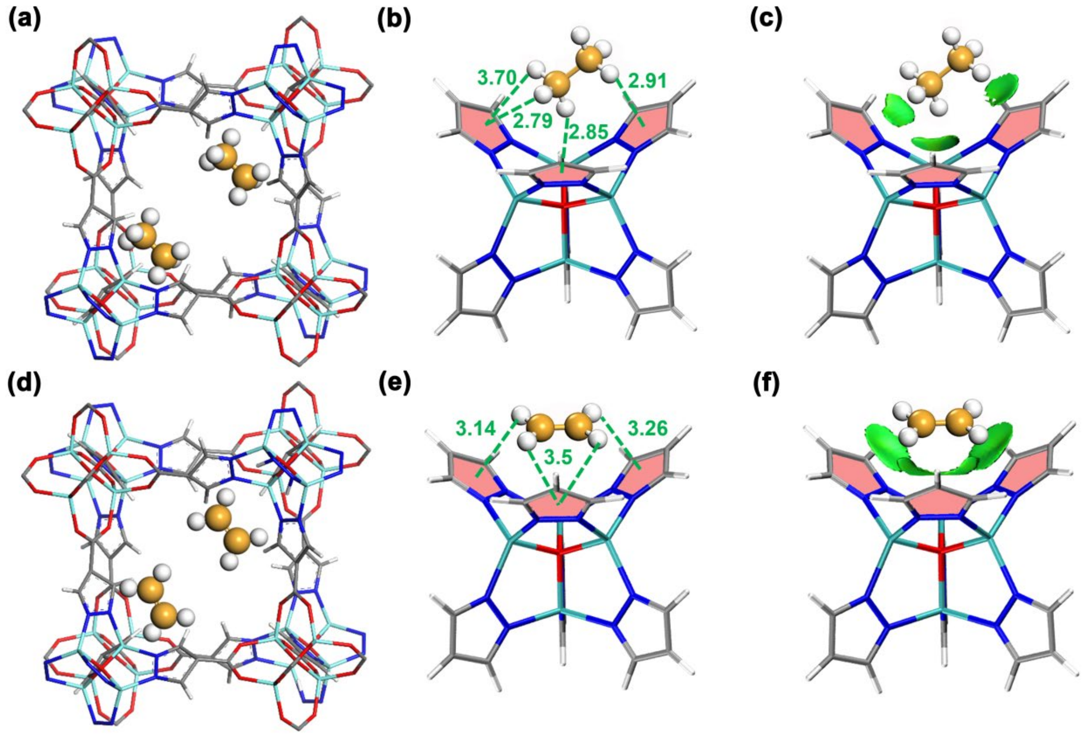

Fig. S28 Primary adsorption sites for C_2H_6 (a) and C_2H_4 (d) in JNU-6 determined by Monte Carlo (GCMC) simulations. C–H···π interactions (green dashed lines) for C_2H_6 (b) and C_2H_4 (e) at the adsorption site of JNU-6. Independent gradient model based on Hirshfeld partition (IGMH) for C_2H_6 (c) and C_2H_4 (f) at the adsorption site of JNU-6 (green surfaces represent vdW interactions). (Color code: Zn, cyan; C, dark gray; N, blue; O, red; H, white. The distance unit is Å).

Table S4. The calculated intermolecular interaction energy between gases and JNU-6 serials of materials.

Parameter	ΔE (C_2H_6) kJ/mol	ΔE (C_2H_4) kJ/mol
JNU-6	-18.04	-17.22
JNU-6-CH_3	-22.23	-20.15

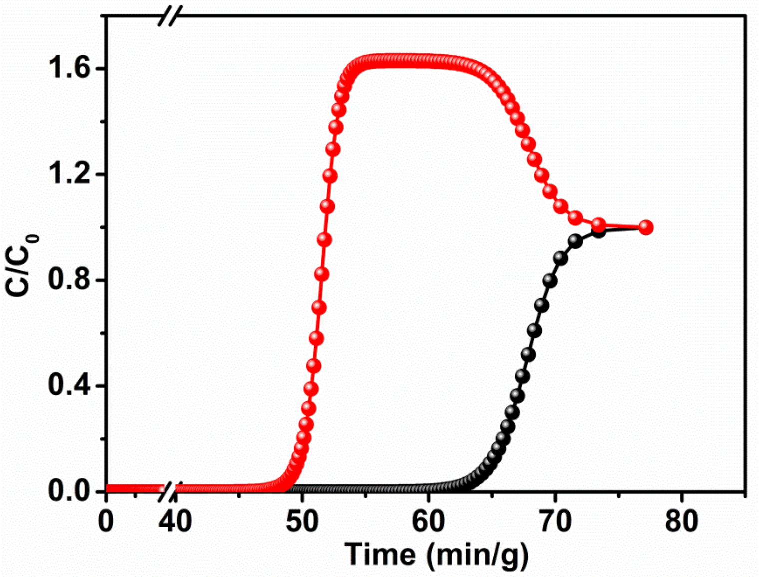

Fig. S29 Transient breakthrough curves for the C_2H_6/C_2H_4 (50:50) mixture in the fixed bed packed with JNU-6-CH_3 at 298 K and 1 bar.

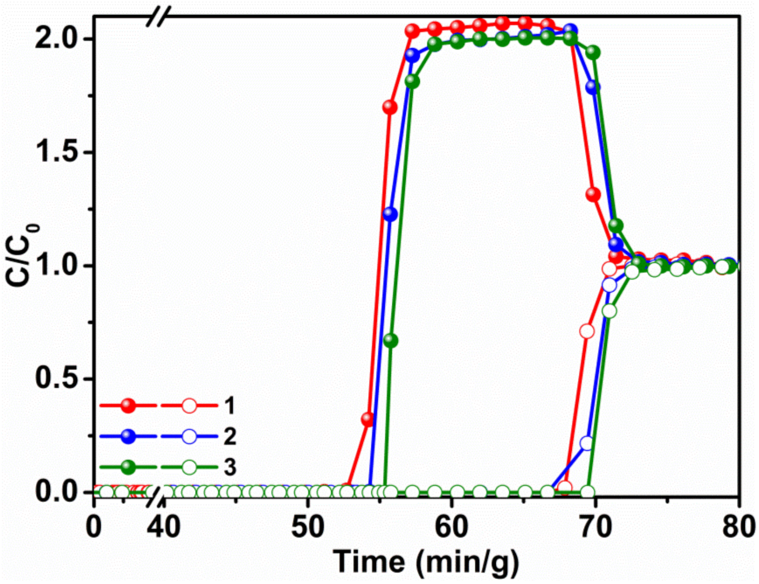

Fig. S30 Three cycles of breakthrough experiments on JNU-6-CH_3 for a C_2H_6/C_2H_4 (50/50, v/v) mixture at a flow rate of 2.0 mL min^{-1} and 298 K under 0% RH conditions.

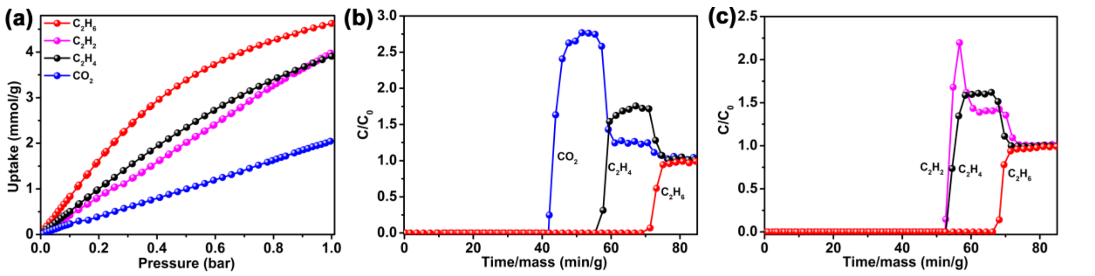

Fig. S31. (a) C_2H_6, C_2H_4, C_2H_2, and CO_2 adsorption isotherms of JNU-6-CH_3 at 298 K. (b) Experimental breakthrough curves of JNU-6-CH_3 (0.70 g) for a $C_2H_6/C_2H_4/CO_2$ (1/1/1, v/v/v) mixture at a flow rate of 2.0 mL min^{-1} and 298 K. (c) Experimental breakthrough curves of JNU-6-CH_3 (0.70 g) for a $C_2H_6/C_2H_4/C_2H_2$ (1/1/1, v/v/v) mixture at a flow rate of 2.0 mL min^{-1} and 298 K. Based on the breakthrough curves, the relative adsorption selectivities of JNU-6-CH_3 were estimated to be 1.7/1.3/1, and 1.3/1.03/1 for $C_2H_6/C_2H_4/CO_2$ (1/1/1, v/v/v), and $C_2H_2/C_2H_4/C_2H_6$ (1/1/1, v/v/v) at 298 K, respectively.

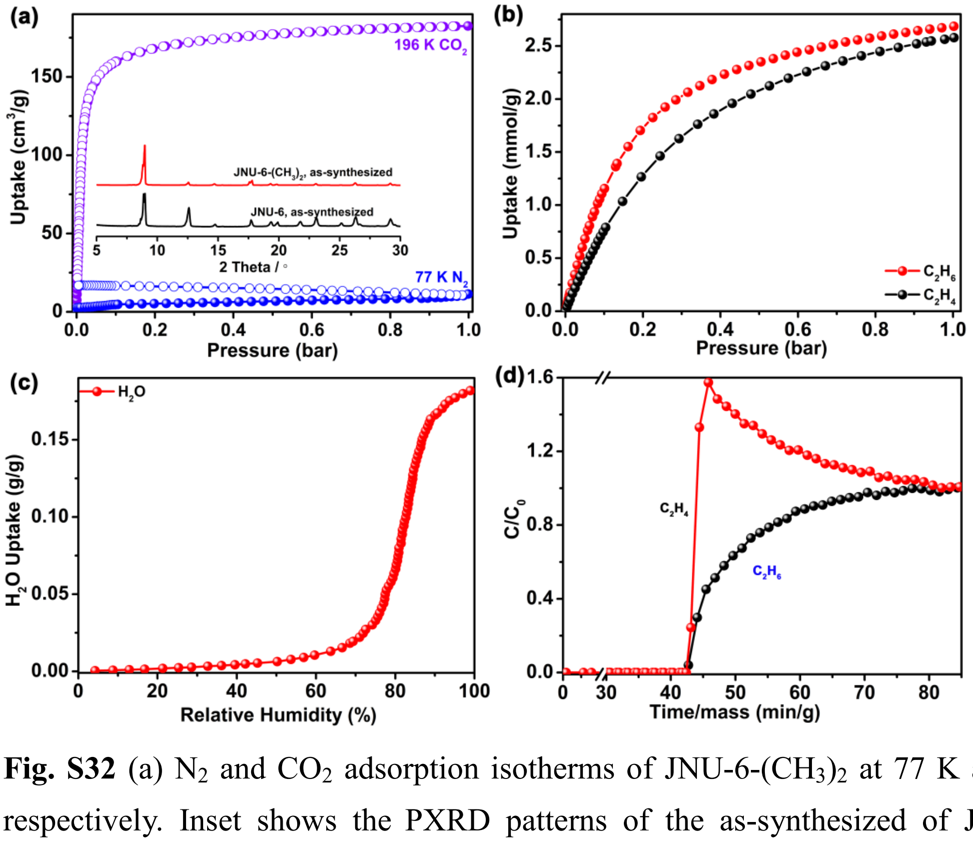

Fig. S32 (a) N_2 and CO_2 adsorption isotherms of JNU-6-$(CH_3)_2$ at 77 K and 196 K respectively. Inset shows the PXRD patterns of the as-synthesized of JNU-6 and JNU-6-$(CH_3)_2$. (b) C_2H_6 and C_2H_4 adsorption isotherms of JNU-6-$(CH_3)_2$ at 298 K (c) Water vapor adsorption isotherm of JNU-6-$(CH_3)_2$ at 298 K. (d) Experimental breakthrough curves on JNU-6-$(CH_3)_2$ (0.92 g) for a C_2H_6/C_2H_4 (50/50, v/v) mixture at a flow rate of 2.0 mL min^{-1} and 298 K under 0% RH conditions.

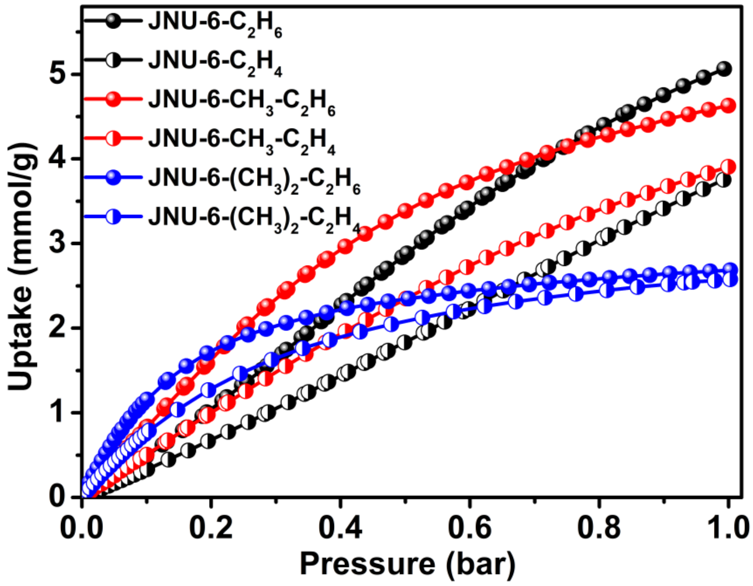

Fig. S33 C_2H_6 and C_2H_4 adsorption isotherms of JNU-6, JNU-6-CH_3, and JNU-6-$(CH_3)_2$ at 298 K.

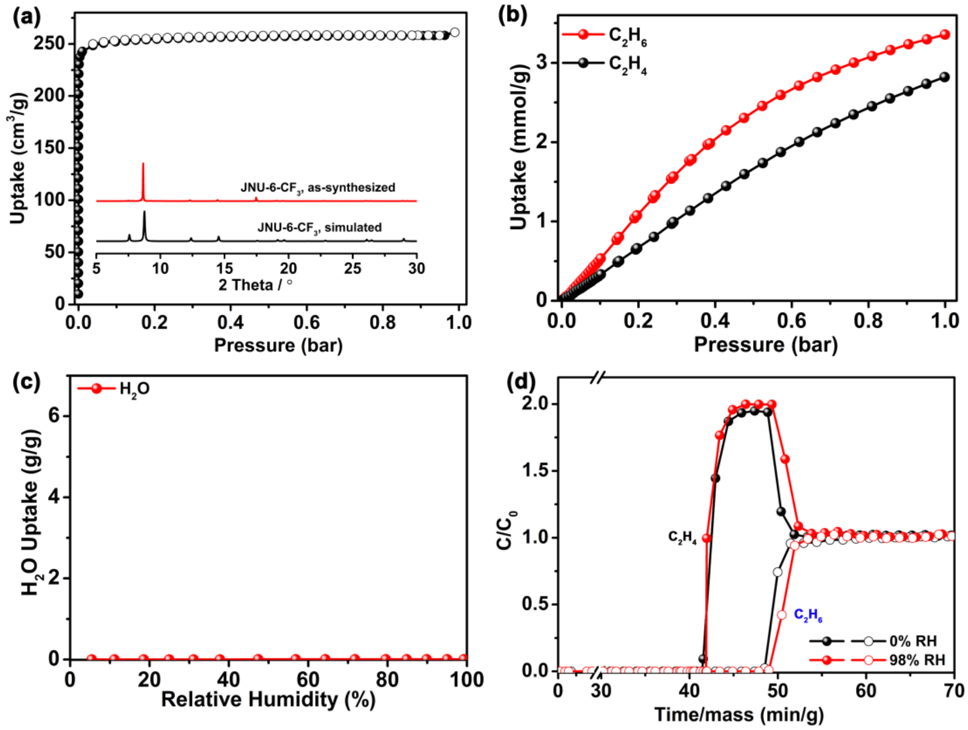

Fig. S34 (a) N_2 adsorption/desorption isotherms of JNU-6-CF_3 at 77 K. Inset shows the PXRD patterns of the as-synthesized and simulated for JNU-6-CF_3. (b) C_2H_6 and C_2H_4 adsorption isotherms of JNU-6-CF_3 at 298 K. (c) Water vapor adsorption isotherm of JNU-6-CF_3 at 298 K. (d) Experimental breakthrough curves of JNU-6-CF_3 (0.9 g) for a C_2H_6/C_2H_4 (50/50, v/v) mixture at a flow rate of 2.0 mL min^{-1} and 298 K under dry or 98% RH conditions. Based on the breakthrough curves, the relative adsorption selectivity of JNU-6-CH_3 was estimated to be 1.3/1 for C_2H_6/C_2H_4 (1/1, v/v).

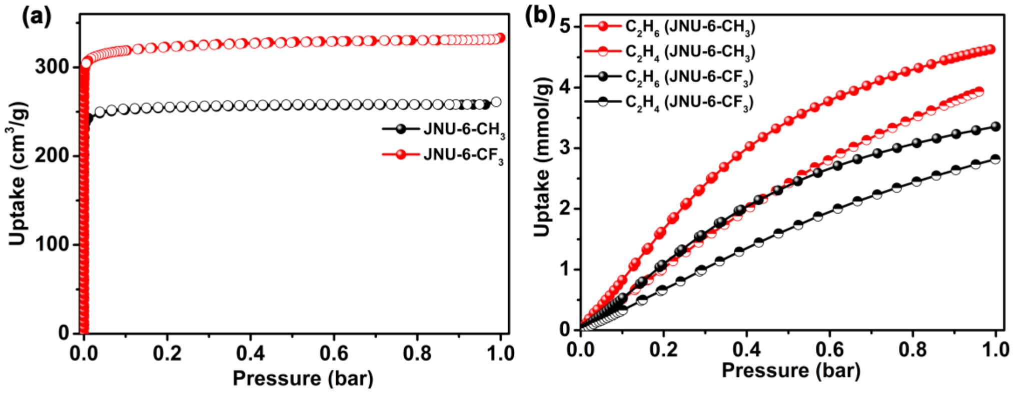

Fig. S35 (a) N_2 adsorption/desorption isotherms of JNU-6-CH_3 and JNU-6-CF_3 at 77 K. (b) C_2H_6 and C_2H_4 adsorption isotherms of JNU-6-CH_3 and JNU-6-CF_3 for at 298 K.

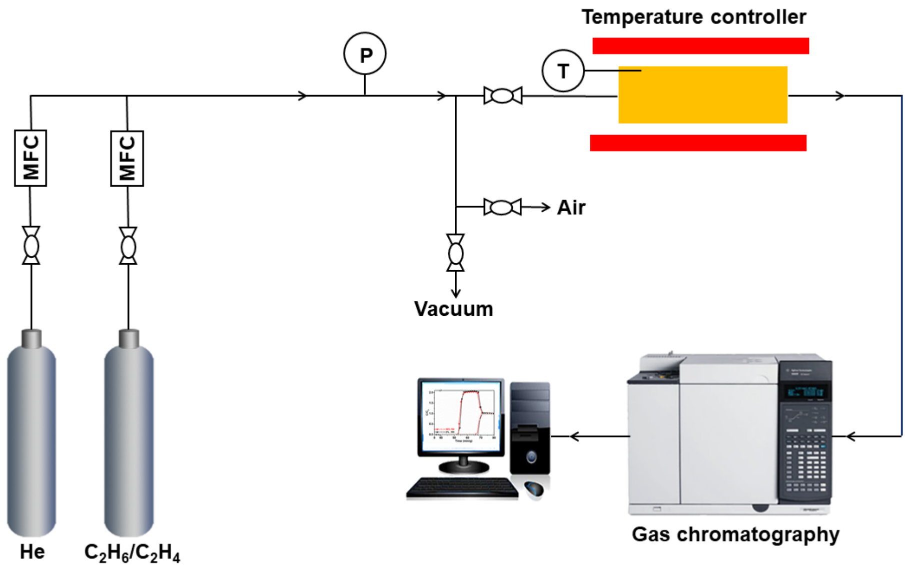

Fig. S36 Schematic illustration of the setup for breakthrough experiments.

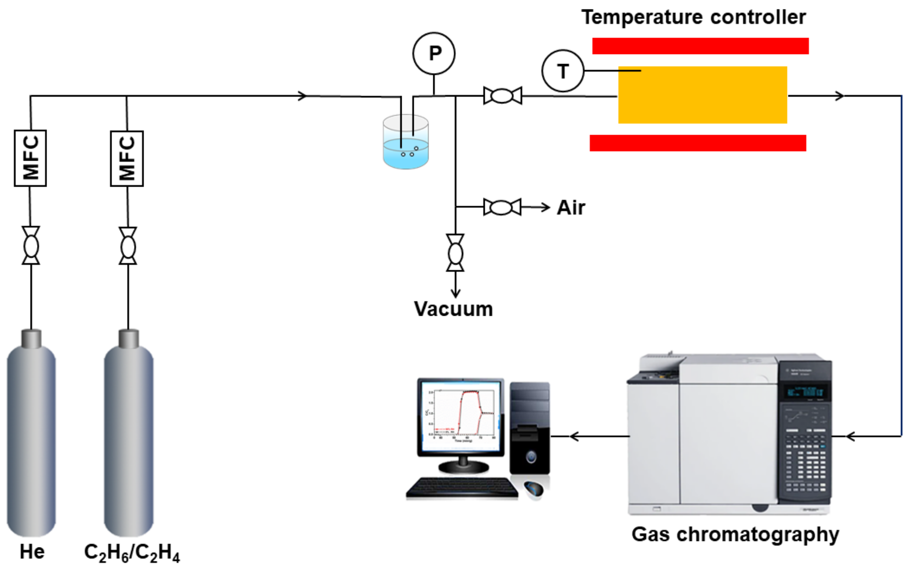

Fig. S37 Schematic illustration of the apparatus for the breakthrough experiments under humid conditions.

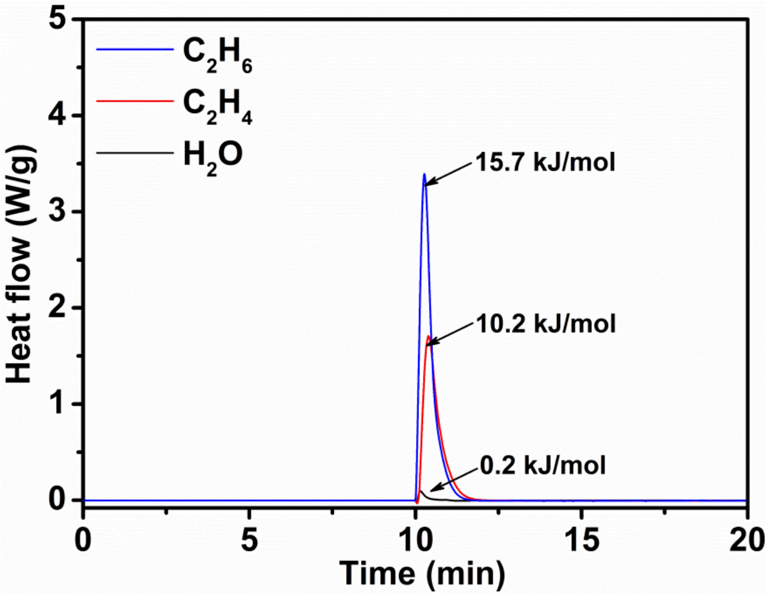

Fig. S38. Differential scanning calorimetry (DSC) for the adsorption of C_2H_6**,** C_2H_4 and $\mathbf{H_2O}$ on JNU-6 at 298 K and 1 bar.

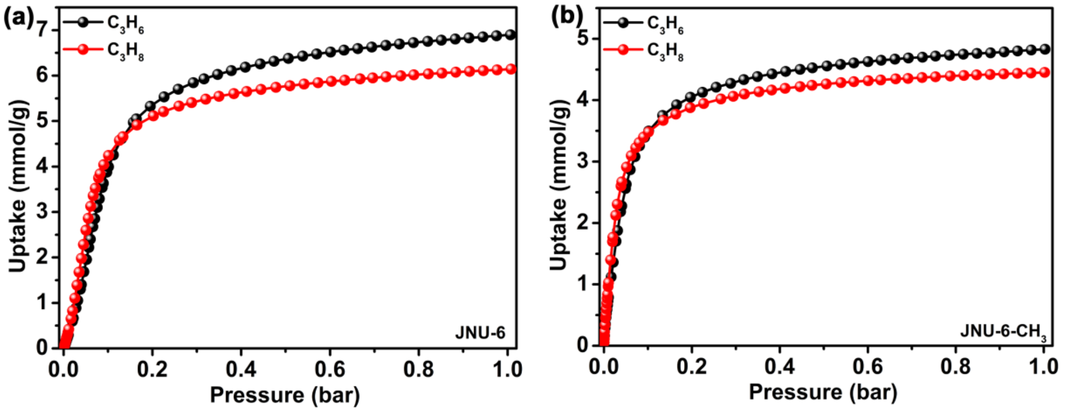

Fig. S39. C_3H_6 and C_3H_8 adsorption isotherms of JNU-6 and JNU-6-CH_3 at 298 K.

Table S5. Crystal data of JNU-6 JNU-6-CH_3 and JNU-6-CF_3.

	JNU-6	JNU-6-CH_3	JNU-6-CF_3
**Formula**	$C_4H_2N_{2.3}Zn$	$C_5H_4N_{2.25}Zn$	$C_{4.5}HF_3N_{2.25}Zn$
**CCDC number**	2259108	2258075	2286047
**Space group**	$Fm\bar{3}c$	$Fm\bar{3}c$	$Fm\bar{3}c$
**Crystal system**	cubic	cubic	cubic
**a (Å)**	20.11	20.15	20.18
**b (Å)**	20.11	20.15	20.18
**c (Å)**	20.11	20.15	20.18
**α (deg)**	90	90	90
**β (deg)**	90	90	90
**γ (deg)**	90	90	90
**V $(Å)^3$**	8140.9 (4)	8184.52 (17)	8226.3 (3)
**Z**	1	1	1
**ρ calcg/cm^3**	1.172	1.256	1.599
**μ/mm^{-1}**	3.019	3.039	3.604
**Final R**	R1= 6.77	R1= 5.52	R1=6.42
**[I>2 sigma (I)]**	wR1=19.84	wR1=15.27	wR1=17.86
**GooF**	1.099	1.098	1.113
**Completeness**	100%	100%	100%

Table S6. Comparison of adsorption capacity, selectivity, and Q_{st} for some selected MOFs.

**MOFs**	**C_2H_6 uptake (mmol/g)**	**C_2H_4 uptake (mmol/g)**	**C_2H_6/C_2H_4 Selectivity**	**Q_{st} C_2H_6/C_2H_4 (kJ/mol)**	**Ref**
JNU-6	5.07	3.77	1.94	17.7/15.7	This work
JNU-6-CH_3	4.63	3.93	2.27	21.2/20.2	This work
NKMOF-8-Br	4.22	3.67	2.65	40.8/33.6	31
NKMOF-8-Me	4.82	4.67	1.88	38.4/37.6	31
Cu $(Qc)_2$	1.85	0.78	3.4	29/25.4	32
IRMOF-8	3.6	2.75	1.6	52.5/50	33
MAF-49	1.72	1.69	2.7	61/48	34
ZIF-7	2.0	1.82	1.5	-/-	35
$Fe_2(O_2)$ (dobdc)	3.4	2.6	4.4	66.8/36.5	36
CPM-733	7.1	6.3	1.75	23.4/22.5	37
JNU-2	4.1	3.6	1.6	29.4/26.7	38
NPU-2	4.42	3.42	1.52	19.6/18.2	39
MUF-15	4.69	4.15	1.96	29.2/28.2	40
MCIF-1	2.4	2.19	1.61	30/29	41
TJT-100	3.66	3.4	1.2	29/25	42
Zn-atz-ipa	1.81	1.8	1.7	45.8/40	43
Ni $(IN)_2$	3.05	0.89	2.44	34.5/33.3	44
AzoleTh-1	4.47	3.62	1.46	28.6/26.1	45
Tb-MOF-76(NH_2)	3.27	2.97	2.05	32.8/22.4	46
FJI-H11-Me(des)	2.59	2.05	2.09	38.9/25.9	47
1a	3.63	3.28	2.15	31.8/23.2	48
UIO-67-$(NH_2)_2$	5.32	4.32	1.7	26.5/24.5	49
Zn-atz-oba	2.1	2	1.27	30/27	50

References

1. A. L. Myers, J. M. Prausnitz, *A.I.Ch.E.J.* 1965, **11**, 121-130.
2. D. Dubbeldam, A. Torres-Knoop, K. S. Walton, *Mol. Simul.*, 2013, **39**, 1253-1292.
3. D. Dubbeldam, S. Calero, D. E. Ellis, R. Q. Snurr, *Mol. Simul.*, 2016, **42**, 81-101.
4. S. L. Mayo, B. D. Olafson, W. A. Goddard, *J. Phys. Chem.*, 1990, **94**, 8897-8909.
5. A. K. Rappé, C. J. Casewit, K. S. Colwell, W. A. Goddard, W. M. Skiff, UFF, *J. Am. Chem. Soc.*, 1992, **114**, 10024-10035.
6. D. Dubbeldam, S. Calero, T. J. H. Vlugt, R. Krishna, T. L. M. Maesen, B. Smit, *J. Phys. Chem. B.*, 2004, **108**, 12301-12331.
7. S. Ban, A. V. Laak, P. E. Jongh, J. P. J. M. Eerden, T. J. H. Vlugt, *J. Phys. Chem. C.*, 2007, **111**, 17241-17248.
8. T. A. Manz, D. S. Sholl, *J. Chem. Theory. Comput.*, 2010, **6**, 2455-2468.
9. G. Kresse, J. Furthmuller, Phys. Rev. B: Condens. *Matter Mater. Phys.*, 1996, **54**, 11169-11186.
10. G. Kresse, D. Joubert, Phys. Rev. B: Condens. *Matter Mater. Phys.*, 1999, **59**, 1758-1775.
11. A. D. Becke, *J. Chem. Phys.*, 1992, **96**, 2155-2160.
12. S. Grimme, J. Antony, S. Ehrlich, H. Krieg, *J. Chem. Phys.*, 2010, **132**, 154104.
13. P. C. Hariharan, J. A. Pople, *Theoretica. Chimica. Acta.*, 1973, **28**, 213-222.
14. L. E. Roy, P. J. Hay, R. L. Martin, *J. Chem. Theory. Comput.*, 2008, **4**, 1029-1031.
15. S. F. Boys, F. Bernardi, *Mol. Phys.*, 1970, **19**, 553-566.
16. M. J. Frisch, G. W. Trucks, H. B. Schlegel, G. E. Scuseria, M. A. Robb, J. R. Cheeseman, G. Scalmani, V. Barone, G. A. Petersson, H. Nakatsuji, Gaussian 16, revision B.01; Gaussian, Inc.: Wallingford, CT, 2016. **66**.
17. J. S. Murray, P. Politzer, Electrostatic potentials: chemical applications. In: Schleyer PvR (ed) Encyclopedia of computational chemistry. Wiley, West Sussex, pp 1998, 912-920.
18. J. Zhang, T. Lu, *Phys. Chem. Chem. Phys.*, 2021, **23**, 20323.
19. T. Lu, Q. J. Chen, *J. Comput. Chem.*, 2022, **43**, 539-555.
20. T. Lu, F. W. Chen, *J. Comput. Chem.*, 2012, **33**, 580-592.
21. W. Humphrey, A. Dalke, K. Schulten, VMD: Visual molecular dynamics, *J. Mol. Graphics.*, 1996, **14**, 33-38.

22. J. Liu, J. Tian, P. K. Thallapally, B. P. McGrail, *J. Phys. Chem. C.*, 2012, **116**, 9575-9581.
23. R. Krishna, *RSC Adv.* **2017**, 7, 35724-35737.
24. R. Krishna, *ACS Omega* **2020**, 5, 16987−17004.
25. R. Krishna, *Microporous Mesoporous Mater.* **2014**, *185*, 30-50.
26. R. Krishna, *RSC Adv.* **2015**, *5*, 52269-52295.
27. R. Krishna, *Sep. Purif. Technol.* **2018**, *194*, 281-300.
28. S. Q. Yang, T. L. Hu. *Coord. Chem. Rev.*, 2022, **468**, 214628.
29. C. Graham, J. Pierrus, R. E. Raab, *Mol. Phys.*, 1989, **67**, 939-955.
30. J. Zhang, T. Lu, *Phys. Chem. Chem. Phys.*, 2021, **23**, 20323-2032.
31. S. B. Geng, E. Lin, X. Li, *J. Am. Chem. Soc.*, 2021, **143**, 8654-8660.
32. R. B. Lin, H. Wu, L Li, X. L. Tang, Z. Li, J. Gao, H. Cui, W. Zhou, B. L. Chen, *J. Am. Chem. Soc.*, 2018, **140**, 12940-12946.
33. J. Pires, M. L. Pinto, V. K. Saini, *ACS Appl. Mater. Interfaces.*, 2014, **6**, 12093-12099.
34. P. Q. Liao, W. X. Zhang, J. P. Zhang, X. M. Chen, *Nat. Commun.*, 2015, **6**, 8697-8705.
35. C. Gucuyener, J. Van den Bergh, J. Gascon, F. Kapteijn, *J. Am. Chem. Soc.*, 2010, **132**, 17704-17706.
36. L. Li, R.-B Lin, R. Krishna, H. Li, S. Xiang, H. Wu, J. Li, W. Zhou, B. Chen, *Science.*, 2018, **362**, 443-446.
37. H. J. Yang, Y. X. Wang, R. Krishna, X. X. Jia, Y. Wang, A. N. Hong, C. Dang, H. E. Castillo, X. H. Bu, P. Y. Feng, *J. Am. Chem. Soc.*, 2020, **142**, 2222-2227.
38. H. Zeng, X. J. Xie, M. Xie, Y. L. Huang, D. Luo, T. Wang, Y. Zhao, W. Lu, D. Li, *J. Am. Chem. Soc.*, 2019, **141**, 20390-20398.
39. B. Zhu, J. Cao, S. Mukherjee, T. Pham, T. Zhang, T. Wang, X. Jiang, K. A. Forrest, M. J. Zaworotko, K. *J. Am. Chem. Soc.*, 2021, **143**, 1485-1492.
40. O. T. Qazvini, R. Babarao, Z-L. Shi, Y-B. Zhang, S. G. Telfer, *J. Am. Chem. Soc.*, 2019, **141**, 5014-5020.
41. N. Zhao, P. Li, X. Mu, C, Liu, F. Sun, G. Zhu, *Faraday. Discuss.*, 2017, **201**, 63-70.
42. H. G. Hao, Y. F. Zhao, D. M. Chen, J. M. Yu, K. Tan, S. Q. Ma, Y. Chabal, Z. M. Zhang, J. M. Dou, Z. H. Xiao, G. Day, H. C. Zhou, T. B. Lu, *Angew. Chem. Int. Ed.*, 2018, **57**, 16067-16071.

43. K. J. Chen, D. G. Madden, S. Mukherjee, T. Pham, K. A. Forrest, A. Kumar, B. Space, J. Kong, Q. Y. Zhang, M. J. Zaworotko, *Science*, 2019, **366**, 241-246.
44. M. Kang, S. Yoon, S. Ga, D.W. Kang, S. Han, J.H. Choe, H. Kim, D.W. Kim, Y.G. Chung, C.S. Hong, *Adv. Sci.*,2021, **8**, 2004940.
45. Z. Xu, X. Xiong, J. Xiong, R. Krishna, L. Li, Y. Fan, F. Luo, B. Chen, *Nat. Commun.*, 2020, **11**, 3163.
46. G. D. Wang, R. Krishna, Y. Z. Li, W. J. Shi, L. Hou, Y. Y. Wang, Z. H. Zhu, *Angew. Chem. Int. Ed.*, 2022, **61**, e202213015.
47. Z. Y. Di, C. P. Liu, J. D Pang, S. X. Zou, Z. Y. Ji, F. L. Hu, C. Chen, D. Q. Yuan, M. C. Hong, M. Y. Wu, *Angew. Chem. Int. Ed.*,2022, **61**, e202210343.
48. G. D. Wang, Y. Z. Li, W. J. Shi, L. Hou, Y. Yu. Wang, Z. H. Zhu, *Angew. Chem. Int. Ed.*, 2022, **61**, e202205427.
49. X.W. Gu, J. X. Wang, E. Wu, H. Wu, W. Zhou, G. Qian, B. Chen, B. Li, *J. Am. Chem. Soc.*, 2022, **144**, 2614-2623.
50. J. W. Cao, S. Mukherjee, T. Pham, Y. Wang, T. Wang, T. Zhang, X. Jiang, H. J. Tang, K. A. Forrest, B. Space, M. J. Zaworotko, K.-J. Chen, *Nat. Commun.*, 2021, **12**, 6507.
